# Specific Dystrophins Selectively Associate with Inhibitory and Excitatory Synapses of the Mouse Cerebellum and their Loss Alters Expression of P2X7 Purinoceptors and Pro-Inflammatory Mediators

**DOI:** 10.1007/s10571-021-01110-6

**Published:** 2021-06-08

**Authors:** Torquil Jackson, Mohsen Seifi, Dariusz C. Górecki, Jerome D. Swinny

**Affiliations:** 1 School of Pharmacy & Biomedical Sciences, University of Portsmouth, St Michael’s Building, White Swan Road, Portsmouth, PO12DT UK; 2 Leicester School of Pharmacy, De Montfort University, Leicester, LE1 9BH UK; 3 Military Institute of Hygiene and Epidemiology, Kozielska 4, 01-001 Warsaw, Poland

**Keywords:** AMPA, NMDA, Delta 2 receptor, Duchenne muscular dystrophy, P2RX7, Neuroinflammation

## Abstract

Duchenne muscular dystrophy (DMD) patients, having mutations of the DMD gene, present with a range of neuropsychiatric disorders, in addition to the quintessential muscle pathology. The neurobiological basis remains poorly understood because the contributions of different DMD gene products (dystrophins) to the different neural networks underlying such symptoms are yet to be fully characterised. While full-length dystrophin clusters in inhibitory synapses, with inhibitory neurotransmitter receptors, the precise subcellular expression of truncated DMD gene products with excitatory synapses remains unresolved. Furthermore, inflammation, involving P2X purinoceptor 7 (P2RX7) accompanies DMD muscle pathology, yet any association with brain dystrophins is yet to be established. The aim of this study was to investigate the comparative expression of different dystrophins, alongside ionotropic glutamate receptors and P2RX7s, within the cerebellar circuitry known to express different dystrophin isoforms. Immunoreactivity for truncated DMD gene products was targeted to Purkinje cell (PC) distal dendrites adjacent to, or overlapping with, signal for GluA1, GluA4, GluN2A, and GluD2 receptor subunits. P2X7R immunoreactivity was located in Bergmann glia profiles adjacent to PC-dystrophin immunoreactivity. Ablation of all DMD gene products coincided with decreased mRNA expression for G*ria2*, G*ria3,* and G*rin2a* and increased GluD2 immunoreactivity. Finally, dystrophin-null mice showed decreased brain mRNA expression of *P2rx7* and several inflammatory mediators. The data suggest that PCs target different dystrophin isoforms to molecularly and functionally distinct populations of synapses. In contrast to muscle, dystrophinopathy in brain leads to the dampening of the local immune system.

**Supplementary Information:**

The online version contains supplementary material available at 10.1007/s10571-021-01110-6.

## Introduction

Duchenne muscular dystrophy (DMD), a neuromuscular disorder caused by the absence of the dystrophin-encoding DMD gene, is frequently associated with a broad range of symptoms of brain origin. One third of DMD patients present with a cognitive deficit but, and patients collectively have reduced IQ scores lower by about 1 SD compared to the general population (Cotton et al. 2001; Snow et al. 2013). Apart from intellectual deficits, patients also present with a variety of neuropsychiatric disorders. These include affective disorders, autism spectrum disorder, attention deficit hyperactivity disorder, obsessive–compulsive disorder, and epilepsy (Banihani, 2015; Caspers Conway, 2015; Filippo et al. 2012; Hendriksen and Vles 2008; Lee et al. 2018; Parisi et al. 2018; Ricotti, 2016). Unfortunately, while these abnormalities significantly exacerbate the disease burden, relatively little progress has been achieved in understanding the neurobiological basis for the neuropsychiatric component of DMD.

The key difficulty arises from the very complex expression pattern of dystrophins in the brain due to extensive alternative promoter usage and alternative splicing, resulting in variants of both the full-length 427 kDa dystrophin (Dp427) and truncated DMD gene products being expressed in different cells of the nervous system (Byers et al. 1993; D'Souza et al. 1995; Doorenweerd, 2017; Gorecki et al. 1992; Lederfein, 1992; Lidov et al. 1995; Naidoo and Anthony 2020; Tinsley et al. 1993). Importantly, dystrophins provide a scaffold for a set of cytoplasmic, membrane-bound, and extracellular proteins, collectively termed the dystrophin-associated protein complex (DAPC) (Waite et al. 2012), which further anchors proteins including receptors and ion channels. These protein interactomes vary depending on the cellular location and the DMD gene product involved (Gawor and Proszynski 2018; Leyva-Leyva et al. 2018), further increasing the complexity. While it has long been established that a significant proportion of brain dystrophin is expressed in postsynaptic compartments (Lidov et al. 1990), identification of individual gene products within functionally specific populations of synapses remains incomplete. The brain expression of Dp427 revealed a complex regional and cell type-specific expression profile (Gorecki et al. 1992; Huard and Tremblay 1992; Lidov et al. 1990; Sekiguchi 2009). At a subcellular level, Dp427 has been shown to be concentrated in inhibitory synapses and involved in the synaptic anchoring of GABA-A receptors (GABA_A_Rs) (Brunig et al. 2002; Knuesel et al. 1999; Krasowska et al. 2014). In contrast, the precise distribution and interactome of the truncated 71 kDa gene product (Dp71) is yet to be fully understood, despite being considered the main dystrophin expressed within the mouse brain (Naidoo and Anthony 2020; Tadayoni et al. 2012). However, convergent molecular, structural, and electrophysiological analyses suggest its enrichment in glutamatergic synapses (Daoud, 2008; Vaillend et al. 1999). In addition, the selective deletion of mouse Dp71, but not other DMD gene products, results in altered hippocampal glutamatergic synapses (Miranda et al. 2011; Miranda, 2009) and impaired cerebellum-mediated cognitive performance consistent with abnormalities of pathways considered crucial for cerebellar development and function (Helleringer, 2018).

Importantly, in addition to neurons, dystrophin is also expressed in glia with dystrophic astrocytes displaying impairments in glutamate clearance, which can initiate neuronal development defects (Patel 2019). In addition, the loss of dystrophin and DAPC from glial end-feet is associated with a leaky blood–brain barrier (BBB) (Lien et al. 2012; Nico 2003). This intrinsic BBB permeability could be further exacerbated by chronic inflammation (Morris et al. 2018), which is a key component of DMD muscle pathology (De Pasquale et al. 2012; Nitahara-Kasahara et al. 2016; Porter, 2003). Yet, changes in the basal immune status of the dystrophic brain are poorly understood (Rae and O'Malley 2016a). A potential common link between dystrophin, cognitive performance, and the immune system is the P2X7 purinoceptor (P2RX7). Indeed, P2RX7s are one of the main drivers of muscle inflammation in DMD (Young, 2015) and have also been shown to mediate inflammatory processes within the nervous system (Browne 2013). Furthermore, the ablation of P2RX7s in Dp427 dystrophic mice alters hippocampal and cortical functions such as improved recognition memory and diminished anxiogenic-like behaviour (Sinadinos, 2015). However, an association between P2RX7s, truncated dystrophin gene products, glutamatergic synaptic pathways, and cerebellar function remains to be identified. Therefore, the aim of this study was to investigate the comparative expression of truncated dystrophin isoforms, alongside P2RX7s and associated inflammatory mediators, using dystrophin gene-deleted mice and dystrophin epitope-specific antibodies. We focused on the cerebellum because of its known contribution to cognitive processes (Schmahmann and Caplan 2006) and impairments in cerebellar development and function are considered central to the DMD brain phenotype (Cyrulnik et al. 2008; Vicari, 2018). Furthermore, Purkinje cells (PC), the major cerebellar output neurons, also express a PC-specific dystrophin isoform, Dp427p (Gorecki et al. 1992). Finally, both inhibitory and excitatory abnormalities, as a result of dystrophinopathy, have been identified in this brain region (Helleringer et al. 2018; Knuesel et al. 1999).

## Materials and Methods

### Animals

The following transgenic mouse models were used in the study:

1) The C57 BL/10ScSn-Dmd^mdx^/J mouse model (Bulfield et al. 1984), referred to here as mdx, lacks the full-length dystrophin isoforms. These mice were bred on a C57 BL/10 background.

2) The C57 BL/6-*Dmd*^Gt(ROSAbgeo)1Mpd^/J mouse model (Wertz and Fuchtbauer 1998), referred to here as mdx^βgeo^, has the reading-frame disruption downstream of exon 63, one that is present in all dystrophins and, therefore, with all isoforms being ablated. Ablation of dystrophin transcripts and protein in this model was fully detailed in (Wertz and Fuchtbauer 1998; Young 2020). These mice breed normally and exhibit no differences in fertility, compared to WT counterparts (Wertz and Fuchtbauer 1998) and our experience. These mice were bred on a C57 BL/6 J background.

3) The eGFP-GFAP mouse model which expresses enhanced green fluorescent protein (eGFP) under the control of the glial fibrillary acidic protein (GFAP) promotor, bred on a FVB/N genetic background (Nolte, 2001), was used to visualise the cytoplasmic compartment of astrocytes in the cerebellum. These mice were kindly provided by Professor Arthur Butt at University of Portsmouth.

4) The *P2rx7*-deficient mouse model is a global knockout for the P2RX7 (Solle 2001), bred on a C57 BL/6 J background.

Wild-type control mice were, in each instance, of the same genetic background as their gene-deleted counterparts.

Adult male mice, aged 2–3 months (coinciding with the active dystrophic process in mice) were used throughout this project. All mice were maintained in-house by University of Portsmouth’s Bioresources Centre, under specific pathogen-free conditions and in a controlled environment (12-h light/dark cycle, 19 °C to 23 °C ambient temperature, and 45% to 65% humidity) with free access to standard chow and water.

### Immunohistochemistry

Animal tissue was fixed by inducing anaesthesia with isoflurane and maintained with pentobarbitone (1.25 mg/kg of bodyweight, IP) and transcardially perfused with 0.9% saline solution for 2 min, followed by 10 min of a fixative consisting of 1% formaldehyde and 15% v/v saturated picric acid in 0.1 M phosphate buffer (PB), pH 7.4. The relatively weak fixation (1%) was deliberately chosen because the majority of the targeted epitopes were on integral membrane proteins. Since the relevant epitopes of such proteins may be masked by formaldehyde cross-linking, this can be minimised by using a lower concentration of fixative solution compared to the standard 4% formaldehyde protocol (Eyre et al. 2012; Lorincz and Nusser 2008). This is particularly applicable to glutamate receptors (Watanabe et al. 1998). After the perfusion, the brains were dissected from the skull and post-fixed over night at room temperature in the same fixative solution. The following day, the brains were rinsed in 0.1 M PB, after which 60 μm sagittal sections were prepared using a VT 1000 vibrating microtome (Leica, Wetzlar, Germany). The sections were thoroughly washed in 0.1 M PB to remove any residual fixative and then stored in a solution containing 0.1 M PB and 0.05% w/v sodium azide until further processing.

A proteolytic antigen retrieval method was used according to (Watanabe et al. 1998) as follows: tissue sections were incubated for 10 min at 37 ºC in 0.1 M PB on a shaking incubator. The PB was removed and replaced with a solution containing 1 mg/ml pepsin (Sigma Aldrich, St Louis, MI, USA) dissolved in 0.2 M HCL for 10 min. Subsequently, sections were washed three times for 10 min in 50 mM TRIS-buffered saline solution containing 0.3% w/v Triton-X-100 (TBS-Tx). To minimise the non-specific binding of secondary antibodies, tissue sections were pre-incubated in a TBS-Tx solution containing 20% normal serum from the species that the secondary antibodies were raised in (Vector Laboratories, Burlingame, CA, USA) for 2 h at room temperature on a horizontal shaker.

Tissue sections were incubated with a cocktail of primary antibodies diluted in TBS-Tx at 4 ºC overnight on a horizontal shaker. Details of the primary antibodies used are provided in Supplementary Information (SI) Table 1. On the following day, tissue sections were washed in TBS-Tx three times for 10 min periods to remove unbound antibodies. Tissue sections were then incubated in a cocktail containing appropriate secondary antibodies targeted at the Fc region of primary antibodies, for 2 h at room temperature on a horizontal shaker. Secondary antibodies were all raised in donkey and conjugated to either Alexa Fluor™ 488 (Invitrogen, Eugene, OR, USA), indocarbocyanine (Cy^TM^3) or DyLight™ 549, or DyLight™ 649 (Jackson Immunoresearch, West Grove, PA, USA), for 2 h. Sections were then washed three times for 10-min periods in TBS-Tx to remove unbound antibodies, mounted on glass microscope slides, air dried, and sealed with glass coverslips using Vectashield™ antifade mounting medium (Vector Laboratories).

### Antibody Specificity

The specificity of all antibodies used in this study has been fully characterised, as detailed in SI Table 1. Method specificity was confirmed by omitting primary antibodies in the reaction sequence, which served to confirm the specificity of secondary antibodies. In the case of double and triple immunolabelling experiments, confirmation of the lack of cross-reactivity by secondary antibodies was tested by reacting a single primary antibody with the full complement of secondary antibodies.

### Microscopy

Tissue samples were examined with a confocal laser-scanning microscope (LSM 880 with AiryScan or LSM 710; Zeiss, Oberkochen, Germany) using a Plan Apochromat 20x (NA 0.8; pixel size 0.42 μm) objective, Plan Apochromat 40 × DIC oil objective (NA 1.3; pixel size 0.29 μm), Plan Apochromat 63 × DIC oil objective (NA 1.4; pixel size 0.13 μm) objective, or a Plan Apochromat 100 × DIC oil objective (NA 1.46; pixel size 0.08 μm). Detection was with either PMT or AiryScan detectors. All images presented represent a single optical section. Images were acquired using sequential acquisition of the different channels to avoid cross-talk between fluorophores. Pinholes were adjusted to 1.0 Airy unit. In all cases where multiple images were captured from the same immunohistochemical reaction, laser power, pinhole, and exposure settings were captured once on tissue from a representative control section and maintained throughout imaging. Images were processed with Zen software (Zeiss) and exported into bitmap images for processing in ImageJ and Adobe Photoshop (Adobe Systems, San Jose, CA, USA). Only brightness and contrast were adjusted for the whole frame, and no part of any frame was enhanced or modified in any way.

### Colocalisation Analyses

The degree of overlap between immunoreactivity for dystrophin-CT, P2RX7, and a range of molecular markers was assessed, according to previously published protocols (Kelly, 2020). Imaging and quantification were performed as follows: using a Plan Apochromatic 100 × DIC oil objective: within three tissue sections per animal (*n* = 5 WT mice), five fields of view were imaged within lobules one, five and 10, in order represent signal throughout the anterior–posterior extent of the cerebellum. Z-stacks consisting of three optical sections were acquired for each field of view. The optical sections were spaced 15 µm apart in the Z-plane in order to prevent overlap of signal across optical sections or individual neurons. The dimensions of each optical section were 42.7 µm × 42.7 µm × 0.7 µm in the X–Y-Z planes. The Coloc2 function in ImageJ was then used to quantify the relative degree of co-occurrence of overlapping pixels, using the Manders’ overlap coefficient (MOC) (Manders et al. 1993), for individual signals. Average values were obtained for tissues sections and then individual animals. The mean ± SEM MOC was computed for N = 5 WT mice. These data are presented in the Results section of the manuscript.

To approximate the degree of potential random colocalisation, the MOC was recomputed using a mirrored view of either the dystrophin or P2RX7 images, by changing their orientation in the horizontal plane. This mirrored MOC was then expressed as a percentage of the original MOC to indicate the potential level of random colocalisation. These mirror image data, alongside all MOC data, are tabulated in SI Table 2.

### Semi-quantitative Analysis of Changes in Ionotropic Glutamate Receptors (iGluR) Subunit Immunoreactivity Between WT and Dystrophin-deficient mice

The purpose of the quantitative cluster analysis was to determine relative changes in the density and area of clusters immunoreactive for specific glutamate receptor subunits between mdx, mdx^βgeo^ and their WT counterparts. Immunohistochemical protocols for each individual glutamate receptor subunit were optimised to ensure optimal signal-to-noise separation. Acquisition parameters for an individual subunit were optimised within the initial field of view. These settings were then identically applied across all fields of view and all tissue sections from WT and dystrophin-deficient mice. A Plan Apochromatic 100 × DIC oil immersion objective was used. Each image represents one optical section of 28.31 µm^2^ in the X–Y plane with the Z-plane adjusted for 1.0 Airy unit. Five fields of view were captured for each imaged lobule per animal. These settings were chosen to enable consistent imaging of immunoreactive puncta-enriching dendritic spines. All fields of view were taken with the centre region approximately equidistant from the PC layer and pial surface. The ImageJ Cluster Analysis tool (open source, https://fiji.sc/) was used to quantify the number of immunoreactive puncta as follows. A micrograph from a WT sample was selected, and an intensity threshold was manually set to separate background signal from immunolabelling. These setting were applied to all images. The micrograph was then converted into a binary image, and a watershed filter was applied to separate adjacent but overlapping puncta. Finally, puncta were quantitatively characterised using the Particle Analysis algorithm.

### Quantitative Reverse-Transcription Polymerase Chain Reaction

Animals were killed by cervical dislocation and tissue homogenates prepared. To prevent degradation of nucleic acid in samples, fresh tissue was dissected and immediately submerged in liquid nitrogen. Total RNA was isolated from the samples using an RNeasy Mini kit (Qiagen, Venlo, Netherlands) according to the manufacturer’s protocol. Briefly, tissue samples were submerged in a lysis buffer and homogenised. Samples were then centrifuged, and supernatants extracted. The supernatants were then mixed with equal volumes of 70% ethanol to prepare RNA for binding to centrifugation columns. Samples were then added to centrifugation columns and washed by centrifugation with two wash buffers. Finally, RNA was eluted by addition of nuclease-free water and centrifuged. Samples were stored at − 80 ºC. The quality and quantity of the extracted RNA in each tissue were examined with a NanoDrop™ spectrophotometer (ThermoFisher Scientific) and then reverse transcribed to provide cDNA templates for PCR and quantitative real-time PCR (qRT-PCR) reactions. Reverse-transcription was achieved by incubating RNA isolates for 2 h at 37 ºC in a cocktail containing: 200 ng RNA in 10% v/v reverse transcription buffer (New England Biolabs, Ipswitch, MA, USA), 5% of Oligo(dT) primers (ThermoFisher Scientific), 5% DNTPs (ThermoFisher Scientific), 2.5% M-MulV reverse transcriptase (Applied Biosystems, Foster City, CA, USA), and 2.5% RiboLock RNase inhibitor (ThermoFisher Scientific) made up to 20 µL with nuclease-free water.

qRT-PCR amplification was performed in 96-well plates in a cocktail containing a TaqMan®-specific mastermix (Roche, Burgess Hill, UK), and TaqMan® probes (see SI Table 3) and performed using a LightCycler® 96 system (Roche). The cycling conditions were 95 ºC for 600 s, followed by 40 cycles of 95 ºC for 15 s and 60 ºC for 60 s. Samples were loaded containing probes for the gene of interest (GOI) and *Gapdh*, which was used as a reference gene for relative mRNA quantification calculations. A standard curve comprising serial dilutions of known RNA concentrations was prepared alongside every sample set and loaded onto the 96-well microplate. All samples were loaded in 10 µL duplicates to reduce technical variability. Standard curves were graphically analysed for reaction efficiency of each primer as well as between gene of interest (GOI) and reference gene, and for pipetting consistency. Assays in which the reaction efficiency was within 90–120%, and similar between GOI and reference genes were analysed. Linear regression of the line produced by a graph of C_t_ against RNA concentration was performed and the relative concentration calculated using the following formulae:

$$ \alpha = \hat{e}((C_{t} - m)/c) $$

$$ \alpha (GOI)/\alpha (Gapdh)\; = \;x $$

where α is the product of the linear regression, *e* is Euler’s number (2.718281828), C_t_; cycle threshold is the cycle number at which the qRT-PCR machine detects fluorescence over a pre-defined threshold, m is the gradient of the standard curve line of best fit, c is the intercept, and x is the calculated level of GOI mRNA relative to *Gapdh* mRNA levels in the sample. This technique was chosen as it allowed the performance of extra quality control steps and reduce any inter-experiment variability. qRT-PCR assays were duplicated or triplicated using samples from separate cohorts of animals and relative mRNA levels pooled for statistical analyses.

### Statistical Analysis

In all cases, GraphPad Prism (GraphPad Software, La Jolla, CA, USA) was used for statistical analyses and graphical presentation of the data. The Shapiro–Wilk test was used to determine the normality of data point distribution within sample groups. Normally distributed data were tested for significance using an unpaired two-tailed Student’s t test. Non-normally distributed data were tested for significance using the Mann–Whitney U test. An alpha level of < 0.05 was used to determine statistical significance. For graphical presentation of quantitative data, bars represent the means and the error bars the SEM.

## Results

### Confirmation of the specificity of dystrophin antibodies

**Fig. 1:**
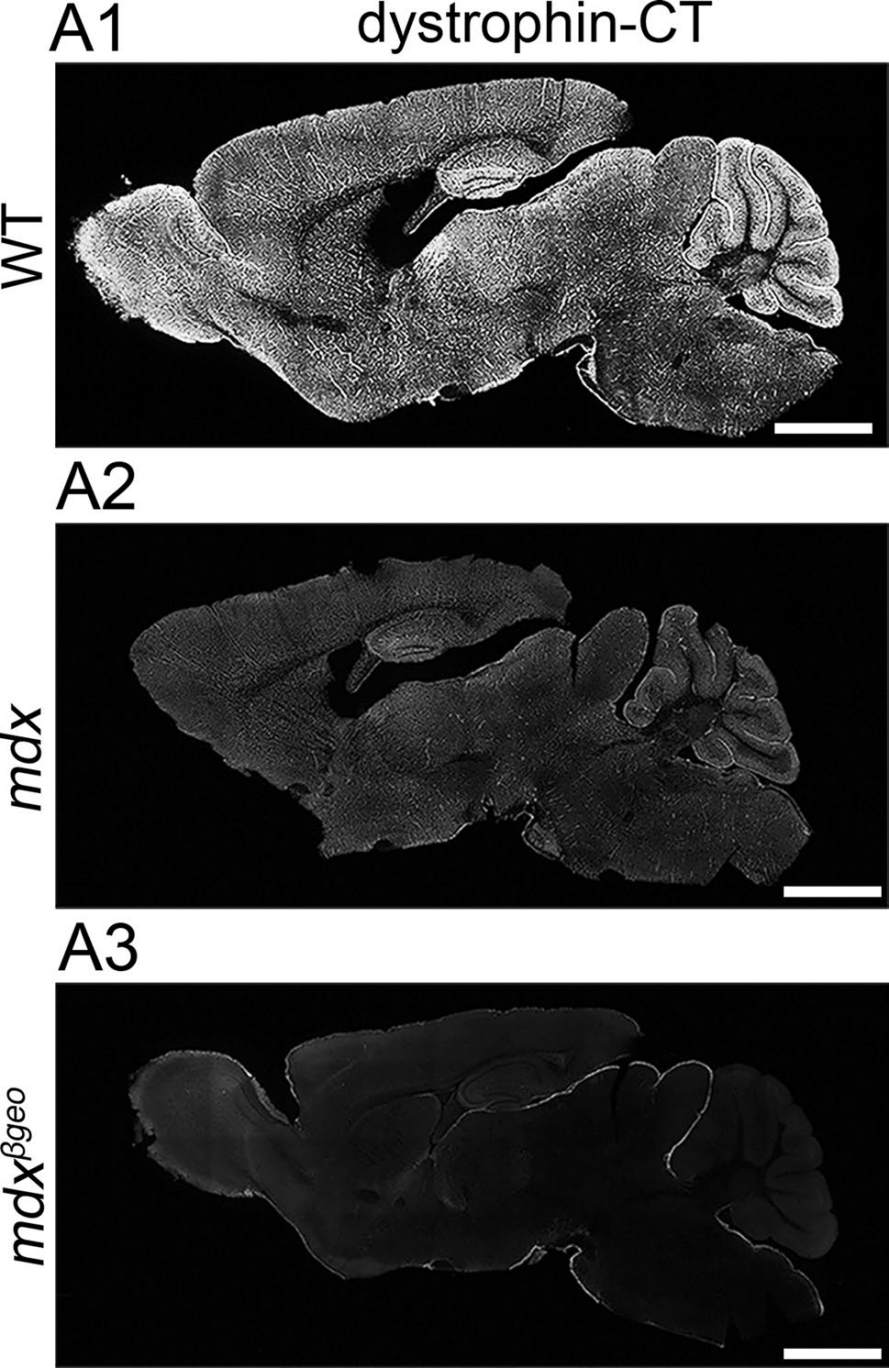
Confirmation of the specificity of dystrophin immunoreactivity and its association with specific subcellular neuronal domains and glial cells of the cerebellar cortex. (A1) low power overview of dystrophin immunoreactivity with an antibody that is targeted to the C-terminus of dystrophin protein (dystrophin-CT) in a sagittal section of a wild type (WT) mouse brain. Signal is widespread throughout the brain, but particularly enriched in the olfactory bulb, dentate gyrus, and cerebellum. (A2) dystrophin-CT immunoreactivity in tissue from the mdx mouse, which lacks the full-length dystrophin isoform (Dp427), reacted, and imaged under conditions identical for (A1) samples. Note the significant decrease in the overall intensity of the signal. (A3) dystrophin-CT immunoreactivity in tissue from the mdx^βgeo^ mouse, which lacks all dystrophin isoforms, reacted, and imaged under conditions identical for (A1) and (A2) samples. Note the absence of any detectable specific signal indicating the specificity of the antibody. Scale bars 1.5 mm

The main anti-dystrophin antibody used throughout the study is directed to an amino acid sequence of the C-terminus of dystrophin (dystrophin-CT), which is common to all dystrophin isoforms. Previously published western blot data for this antibody indicate that in the cerebellum and hippocampus, it recognises the full-length dystrophin, as well as the truncated 140 kDa (Dp140) and Dp71 dystrophins (Hendriksen, 2016). In this study, the dystrophin-CT antibody produced strong signal throughout the brain of WT mice, with particular enrichment noticeable in the olfactory bulb, dentate gyrus, and cerebellum (Fig. 1 A1). In mdx mice, which lack Dp427, there was a noticeable decrease in the intensity of dystrophin-CT immunoreactivity, reacted, and imaged under conditions identical to WT tissue (Fig. 1 A2). In contrast, no specific dystrophin-CT signal was detected in tissue from mdx^βgeo^ mice, which lack all dystrophins (Fig. 1 A3).

**Fig. 2:**
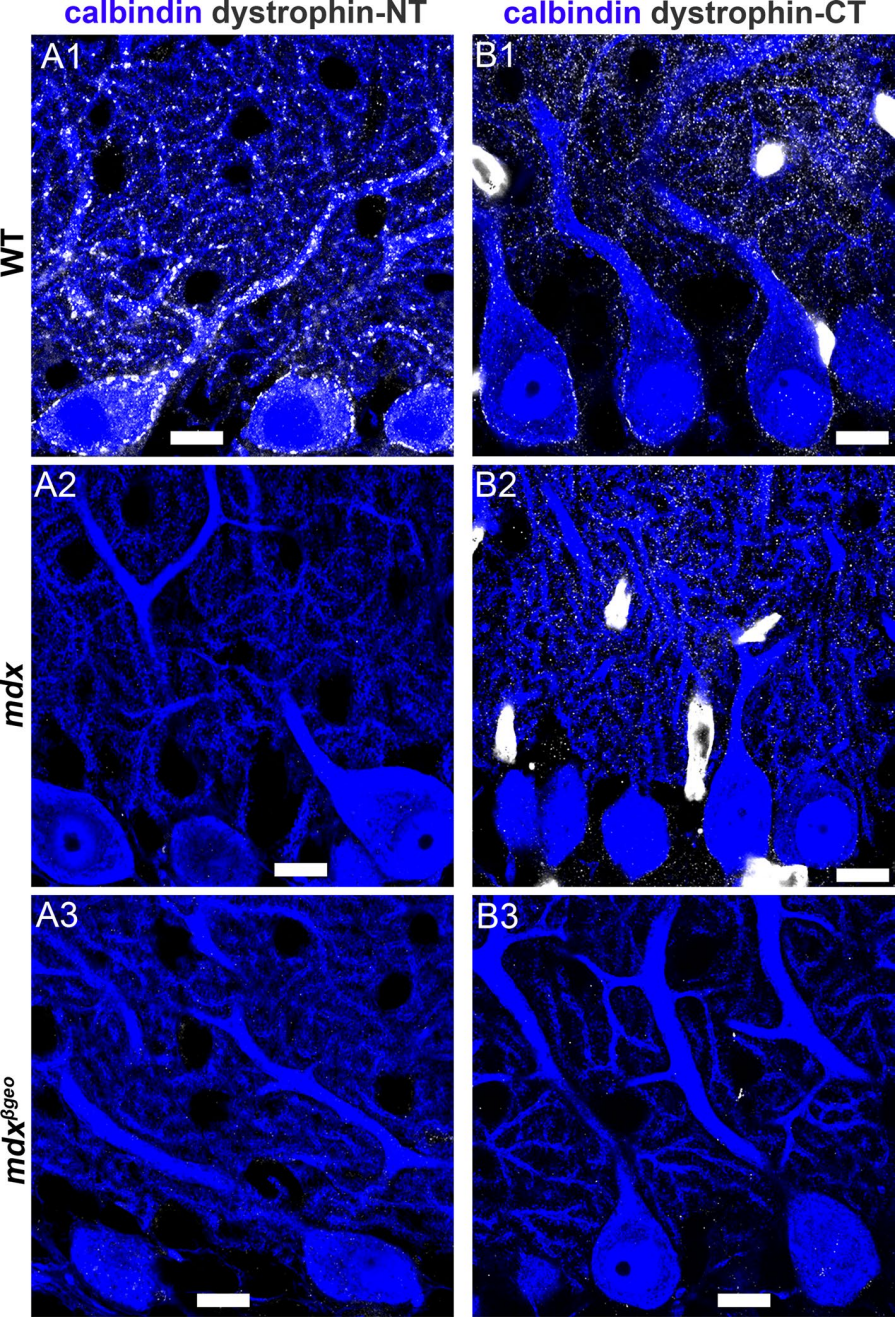
Association of dystrophin immunoreactivity with specific subcellular neuronal domains of the cerebellar cortex. (A1) shows immunoreactivity for dystrophin with an antibody that is targeted to the N terminus of Dp427 (dystrophin-NT) in a sagittal section of the cerebellum of a WT mouse. Individual clusters are concentrated on somatic and proximal dendritic regions of Purkinje cells (PC), identified by immunoreactivity for calbindin. No specific dystrophin-NT signal was detected in samples from (A2) mdx and (A3) mdx^βgeo^ mice. (B1) shows dystrophin-CT immunoreactivity in the molecular layer of a WT mouse. Signal is located on the somatic and proximal dendritic domains of PCs, as well as on their distal dendrites, in addition to blood vessels. (B2) dystrophin-CT immunoreactivity in the cerebellum of an mdx mouse shows the ablation of signal from PC somatic and proximal dendritic regions, with signal in dendritic regions and blood vessels persisting. (B3) no specific dystrophin-CT immunoreactivity is detected in the cerebellum of mdx^βgeo^ mice. Scale bars 10 µm

Previous reports indicate that Dp427 is expressed at inhibitory synapses on PCs, where it anchors GABA_A_Rs (Brunig et al. 2002; Knuesel et al. 1999). We, therefore, first sought to replicate this immunoreactivity pattern using antibodies directed to either the N terminus of Dp427 (dystrophin-NT antibody), or the C-terminus, using the dystrophin-CT antibody, since it recognises all dystrophins in the cerebellum (Hendriksen et al. 2016). In tissue from WT mice, dystrophin-NT immunoreactivity was predictably restricted to the somatic and proximal dendritic regions of PCs (Fig. 2 A1), in a manner similar to the pattern presented in (Knuesel et al. 1999), and where inhibitory synapses are concentrated in this cell type. No specific dystrophin-NT signal was detected in tissue from mdx mice (Fig. 2 A2) nor mdx^βgeo^ mice (Fig. 2 A3). This suggests that signal from the dystrophin-NT antibody, located in inhibitory synaptic domains, corresponds to full-length dystrophin.

Immunoreactivity for dystrophin-CT presented as two distinct patterns of labelling. Apart from labelling blood vessels, signal was localised to not only the somatic compartments in a manner similar to the dystrophin-NT antibody, but also throughout the molecular layer where excitatory synapses predominate (Fig. 2 B1). However, in contrast to the dystrophin-NT antibody, while dystrophin-CT labelling in mdx mice was ablated in somatic and proximal dendritic compartments where inhibitory synapses are predominantly located, signal persisted in distal dendritic regions of the molecular layer which contain excitatory synapses (Fig. 2 B2). Only residual, non-specific dystrophin-CT signal was detectable in tissue from mdx^βgeo^ mice (Fig. 2 B3). This step-wise loss of dystrophin labelling patterns correlating with cumulative loss of dystrophins suggests a differential expression of isoforms at specific PC subcellular compartments. Furthermore, these data indicate that putative-truncated DMD gene products are likely to be preferentially expressed in the vicinity of excitatory synapses and blood vessels, in line with previous evidence (Daoud et al. 2008; Haenggi et al. 2004).

### Putative Short Dystrophins are Associated with Neurons and Glia in the Molecular Layer of the Cerebellar Cortex

**Fig. 3:**
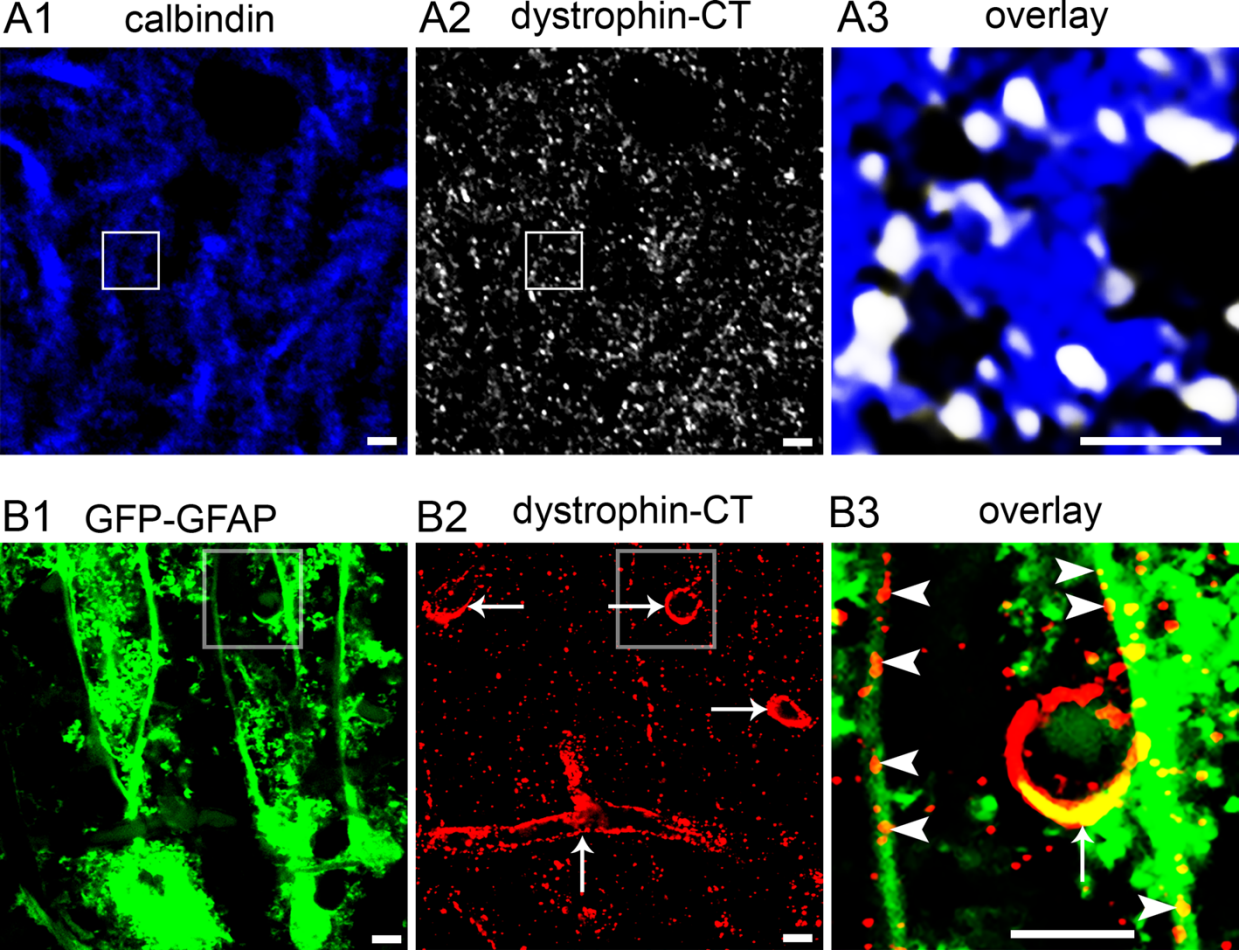
(A) high-resolution image of dystrophin-CT immunoreactivity indicating that the signal is concentrated as individual clusters in the necks and heads of PC dendritic spines, identified by calbindin immunoreactivity. (A3) is a magnified view of the boxed area in (A1) and (A2). (B1) visualisation of Bergmann glia processes in the cerebellum, using green fluorescence protein (GFP) signal, the expression of which is driven by the promotor for glial fibrillary acid protein (GFAP). (B2) in the same field of view, dystrophin-CT immunoreactivity presents as individual clusters as well as profiles reminiscent of blood vessels (arrows). (B3) is a magnified view of the boxed areas in (B1 and 2) indicating the dystrophin-CT immunoreactive clusters located on GFP-GFAP profiles which are likely Bergmann glia shafts (arrowheads), as well as lining a putative blood vessel (arrow). Scale bars: (A) 2 µm; (B) 5 µm

Expression of short dystrophins is reportedly associated with both neuronal and glial elements in the brain (Daoud et al. 2008; Haenggi and Fritschy 2006). High-resolution imaging of dystrophin-CT immunoreactivity within the molecular layer revealed individual clusters (Fig. 3 A1-2) located either in the neck or the head of dendritic spines (Fig. 3 A3). Since PC dendritic spines are ensheathed by Bergmann glia processes, such signal, at this resolution could be representative of expression within such glial networks. To explore this further, we used tissue from mice expressing enhanced green fluorescent protein (eGFP) under the control of the glial fibrillary acidic protein (GFAP) promotor, which in the cerebellum reveals astrocytes, and Bergmann glia, in particular. The benefit of such a tool is that the eGFP signal fills the cytoplasmic compartments of Bergmann glia in their entirety, thus, allowing for the visualisation of the full extremities of these cells. As expected, eGFP-GFAP signal presented as a network of processes contacting PCs around their somata and dendrites (Fig. 3 B1). Clusters immunoreactive for dystrophin-CT associated with GFAP-eGFP signal was in the form of clusters on Bergmann glia shafts as well the periphery of putative blood vessels (Fig. 3 B2-3). A 3D volumetric reconstruction of the region in (Fig. 3B), demonstrating the close association between dystrophin-CT and glial processes is provided as a SI media file (**LINK**). This suggests that the putative short dystrophins are expressed by PCs and glia. In addition, since Bergmann glial processes are known to ensheath excitatory synapses on PC spines, this further suggests the expression of short dystrophins in the close vicinity of glutamatergic synapses.

### Putative Full-Length and Truncated Dystrophins are Differentially Localised to Inhibitory and Excitatory Synaptic Sites, Respectively

**Fig. 4:**
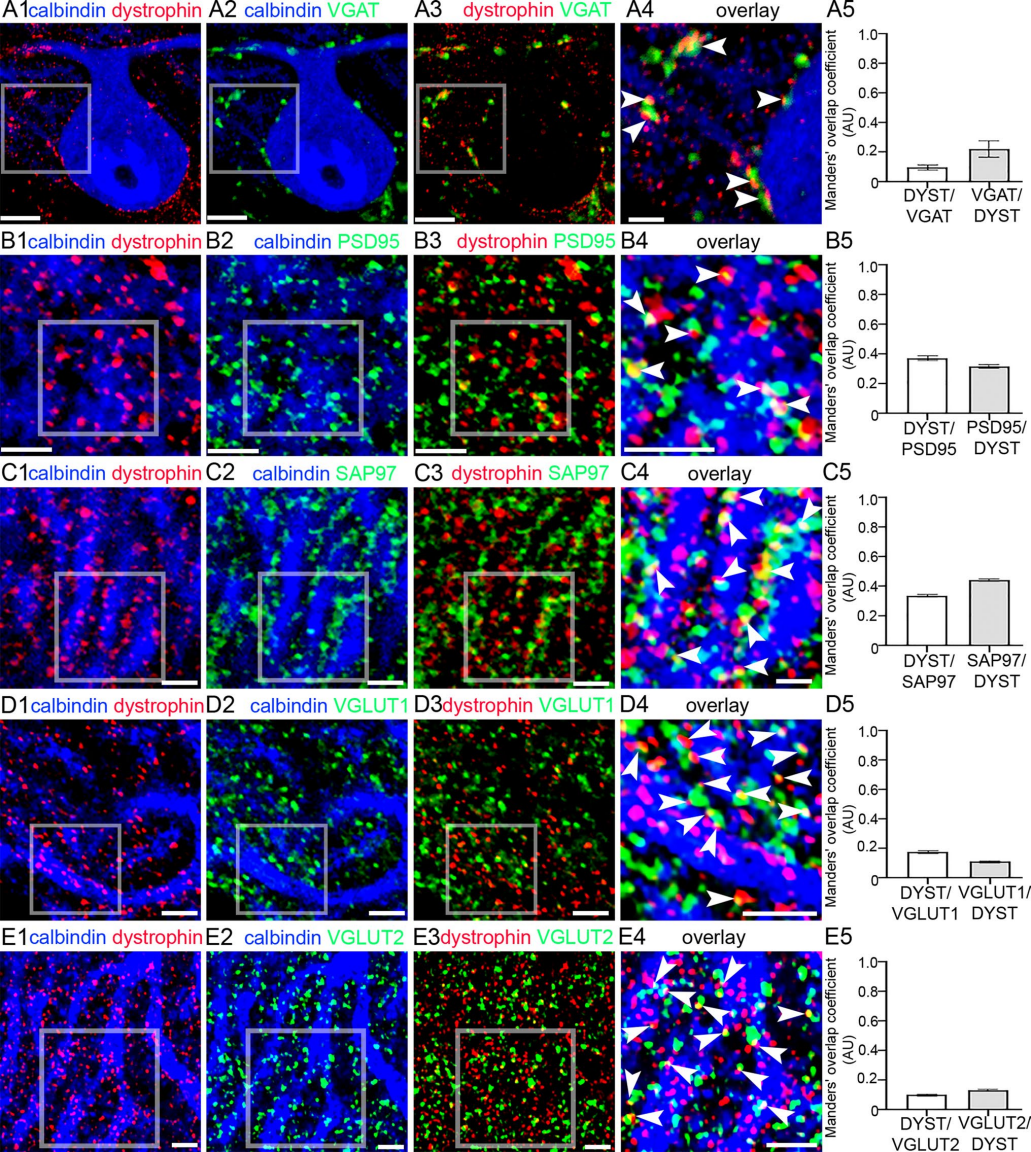
Different dystrophin isoforms are targeted to inhibitory and excitatory synapses in WT mice. (A1–4) Dystrophin-CT immunoreactive clusters are closely associated with puncta immunoreactive for the vesicular GABA transporter (VGAT) (arrowheads), a marker of inhibitory axon terminals. Note that this somatic-proximal dendritic proportion of dystrophin-CT immunoreactivity was ablated in the mdx mouse, which lacks the full- length (Dp427) dystrophin isoform (Fig. 2 B2). This suggests that Dp427 is targeted to inhibitory synapses. (A5) quantification of the relative overlap of dystrophin-CT signal with VGAT, and VGAT with dystrophin-CT, according to the Manders’ overlap coefficient method. The bars represent the means, and the errors the SEM. *N* = 5 animals. (B1–4) dystrophin-CT immunoreactivity within the distal molecular layers is located on PC dendrites adjacent (arrowheads) to, and partially overlaps with, clusters immunopositive for postsynaptic density 95 (PSD-95), a protein integral for the trafficking of glutamate receptors to excitatory synapses and is, thus, indicative of the postsynaptic locations of such receptors and synapses. (B5) quantification of the relative overlap of dystrophin-CT signal with PSD-95, and PSD-95 with dystrophin-CT, according to the Manders’ overlap coefficient method. The bars represent the means, and the errors the SEM. *N* = 5 animals. (C1–4) shows that dystrophin-CT immunoreactivity also is located adjacent to, or partially overlaps with (arrowheads), clusters immunopositive for synapse-associated protein-97 (SAP97), a protein that is integral in the trafficking of glutamate receptors to excitatory synapses and is, thus, indicative of the postsynaptic locations of such receptors and synapses. (C5) quantification of the relative overlap of dystrophin-CT signal with SAP97, and SAP97 with dystrophin-CT, according to the Manders’ overlap coefficient method. The bars represent the means, and the errors the SEM. *N* = 5 animals. (D1**–**4) Proportion of dystrophin-CT immunoreactive clusters is located in close apposition (arrowheads) to puncta immunoreactive for the vesicular glutamate transporter 1 (VGLUT1), a marker of the presynaptic domains of parallel fibres in the cerebellum. (D5) quantification of the relative overlap of dystrophin-CT signal with VGLUT1 and VGLUT1 with dystrophin-CT, according to the Manders’ overlap coefficient method. The bars represent the means, and the errors the SEM. *N* = 5 animals. (E1–4) Proportion of dystrophin-CT immunoreactive clusters is located in close apposition (arrowheads) to puncta immunoreactive for the vesicular glutamate transporter 2 (VGLUT2), a marker of the presynaptic domains of climbing fibres in the cerebellum. (E5) quantification of the relative overlap of dystrophin-CT signal with VGLUT2, and VGLUT2 with dystrophin-CT, according to the Manders’ overlap coefficient method. The bars represent the means, and the errors the SEM. *N* = 5 animals. Scale bars: (A1–3) 5 µm; (A4) 2 µm; (B) 3 µm; (C1–3) 2 µm; (C4) 1 µm; (D1–3) 2 µm; (D4) 1 µm; (E) 2 µm

The patterns of labelling corresponding to putative full-length and truncated dystrophins in Fig. 2 appeared to coincide with the locations of inhibitory and excitatory synapses, respectively. To verify this, we assessed signal for the dystrophin-CT antibody alongside molecular markers of inhibitory and excitatory synapses, in tissue from WT mice. Dystrophin-CT immunoreactive clusters, on somatic and proximal dendritic compartments of PCs, were located in close association with terminals immunopositive for the vesicular GABA transporter (VGAT), a protein expressed exclusively in the membranes of synaptic vesicles which contain either GABA or glycine, and is, thus, predictive of the release sites for these inhibitory neurotransmitters (Fig. 4 A 1–4). Manders’ overlap coefficient (MOC) analysis (Manders et al. 1993) was performed to assess the degree of colocalised pixels between dystrophin-CT and VGAT, and vice versa. This measure quantifies the proportion of co‐occurrence of overlapping pixels between two signals and ranges between 0 and 1. The MOC for dystrophin-CT signal overlapping with VGAT was 0.10 ± 0.02, *N* = 5 animals. The MOC for VGAT signal overlapping with dystrophin-CT was 0.22 ± 0.06 (Fig. 4 A5). This low association with this presynaptic markers confirms the predominant postsynaptic location of dystrophin.

Since this somatodendritic dystrophin-CT signal was ablated in the full-length Dp427-null mouse (mdx) (Fig. 2 B2), this confirms previous reports that full-length dystrophin expression is most likely restricted to inhibitory synapses. However, a proportion of dystrophin-CT immunoreactive clusters, presumably representing the expression of short isoforms, was also associated with pre- and postsynaptic markers of excitatory synapses, within PC distal dendritic domains. Dystrophin-CT immunoreactive clusters were also located in close proximity to, or partially overlapped with, puncta immunopositive for postsynaptic density 95 (PSD-95), a synaptic scaffold protein that regulates the trafficking and localisation of glutamate receptors (Huganir and Nicoll 2013) (Fig. 4 B1-4). The MOC for dystrophin-CT signal overlapping with PSD-95 was 0.37 ± 0.02, *N* = 5 animals. The MOC for PSD-95 signal overlapping with dystrophin-CT was 0.32 ± 0.01 (Fig. 4 B5). Signal for dystrophin-CT was also located either adjacent to, or overlapping with immunoreactivity for synapse-associated protein-97 (SAP97), another postsynaptic scaffolding and trafficking protein strongly associated with glutamate receptors (Fig. 4 C1-4). The MOC for dystrophin-CT signal overlapping with SAP97 was 0.34 ± 0.01, *N* = 5 animals. The MOC for SAP97 signal overlapping with dystrophin-CT was 0.44 ± 0.01 (Fig. 4 C5). Further evidence of the association of truncated dystrophins with glutamatergic synapses was the location of dystrophin-CT immunoreactive clusters in apposition to puncta immunoreactive for vesicular glutamate transporter 1 (VGLUT1) (Fig. 4 D1-4) and vesicular glutamate transporter 2 (VGLUT2) (Fig. 4 E1-4), which are located preferentially within two glutamatergic afferent systems for PCs, namely parallel and climbing fibres, respectively. The MOC for dystrophin-CT signal overlapping with VGLUT1 was 0.18 ± 0.01, *N* = 5 animals, while the MOC for VGLUT1 signal overlapping with dystrophin-CT was 0.11 ± 0.001 (Fig. 4 D5). The MOC for dystrophin-CT signal overlapping with VGLUT2 was 0.10 ± 0.007, *N* = 5 animals, while the MOC for VGLUT2 signal overlapping with dystrophin-CT was 0.13 ± 0.005 (Fig. 4 E5). Thus, collectively, a larger of proportion of dystrophin-CT signal was preferentially associated with post-, rather than presynaptic marker proteins for glutamate synapses.

Since this quotient of dystrophin-CT labelling was ablated in the dystrophin-null mdx^βgeo^ mouse model, but present in the Dp427-null mdx mouse model (Fig. 2), this suggests that short DMD gene products are targeted to glutamatergic synapses.

### Immunoreactivity for Truncated Dystrophins is Located in Close Association with Diverse Glutamate Receptor Subtypes

**Fig. 5:**
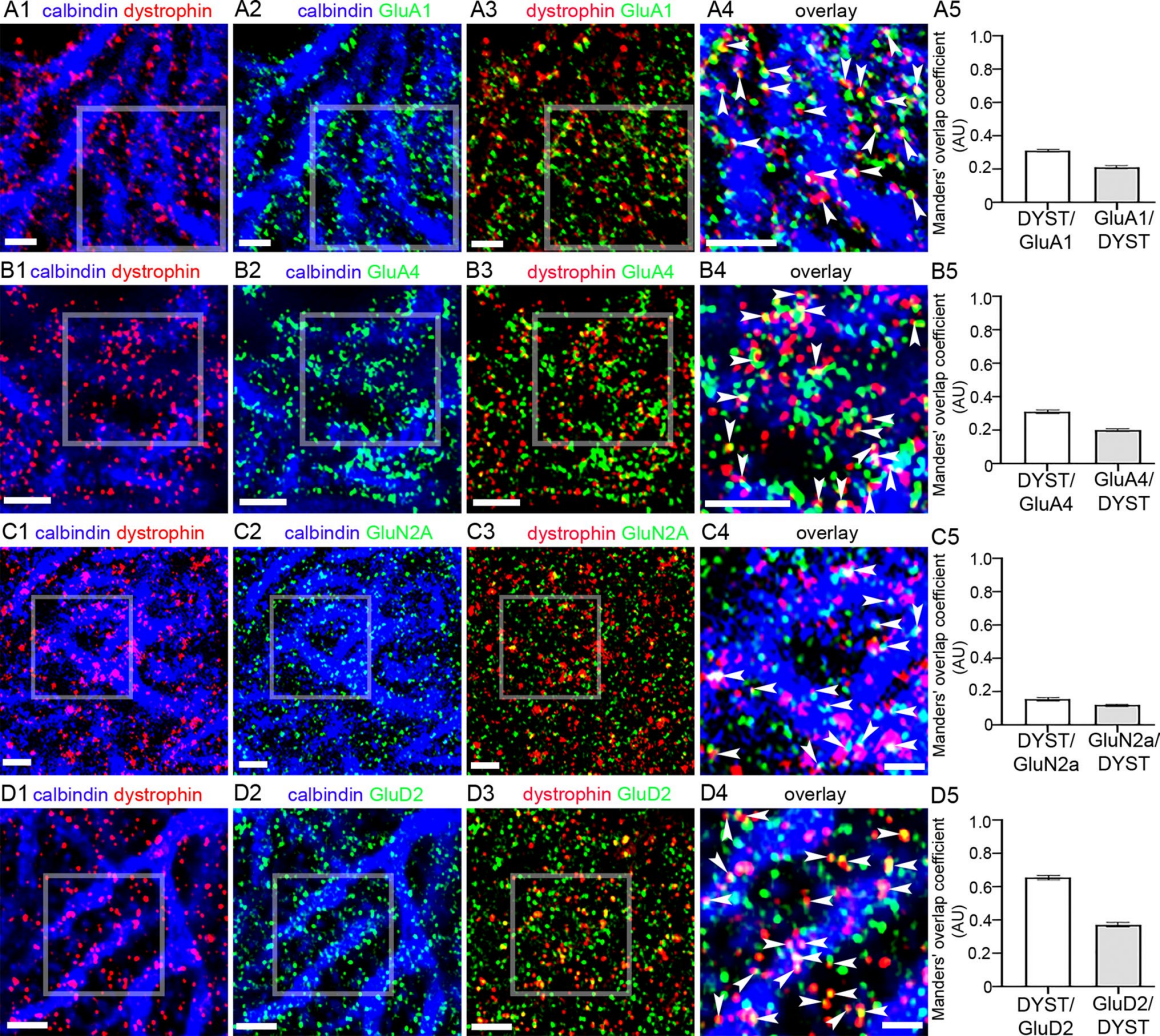
Dystrophin is expressed in close association with specific ionotropic glutamate receptor subunits. A proportion of dystrophin-CT immunoreactive clusters are located adjacent to, or overlap with (arrowheads), clusters immunoreactive for the (A1–4) GluA1, (B1–4) GluA4, (C1–4) GluN2A, and (D1–4) GluD2 subunits. (A–D 5) show quantification of the relative overlap of dystrophin-CT signal with GluA1, GluA4, GluN2A, and GluD2, respectively, according to the Manders’ overlap coefficient method. The bars represent the means, and the errors the SEM. *N* = 5 animals. Scale bars: (A) 3 µm: (B 1–3) 5 µm: (B4) 3 µm; (C) 3 µm; (D1–3) 5 µm; (D4) 3 µm

Given the above location of a proportion of dystrophin-CT immunoreactive clusters with that of molecular markers of glutamatergic synapses, we next explored whether signal for such putative-truncated dystrophin was associated with immunoreactivity for AMPA and NMDA receptor subunits. An important technical caveat is that the proteolytic antigen retrieval method essential for the visualisation of membrane-bound AMPA and NMDA receptor subunits (Watanabe et al. 1998) also results in the ablation of cytoplasmic signal, such as calbindin immunoreactivity, especially in cellular compartments with low levels of such signal such as dendritic spines. This can create the impression that AMPA, NMDA, and dystrophin signal is adjacent to, but not within PCs, potentially in neighbouring glial elements. However, numerous independent studies, using ultrastructural analyses, have unequivocally demonstrated the expression of glutamate receptors in postsynaptic compartments of PCs (Masugi-Tokita et al. 2007; Tanaka, 2005; Zhao et al. 1998). A further technical clarification is that the co-analysis of AMPA subunits with the dystrophin-CT antibody was restricted to the GluA1 (Fig. 5 A) and GluA4 (Fig. 5 B) subunits since available, validated antibodies for the GluA2 and 3 subunits were raised in the same species. Immunoreactivity for both GluA1 (Fig. 5 A2) and GluA4 (Fig. 5 B2) was located in PCs, in agreement with previous reports. Clusters immunoreactive for dystrophin-CT frequently overlapped with or were in close apposition to GluA1 (Fig. 5 A3-4) or GluA4 (Fig. 5 B3-4) immunoreactive clusters. The MOC for dystrophin-CT signal overlapping with GluA1 was 0.31 ± 0.01, *N* = 5 animals, while the MOC for GluA1 signal overlapping with dystrophin-CT was 0.21 ± 0.01 (Fig. 5 A5). The MOC for dystrophin-CT signal overlapping with GluA4 was 0.31 ± 0.01, *N* = 5 animals, while the MOC for GluA4 signal overlapping with dystrophin-CT was 0.20 ± 0.01 (Fig. 5 B5).

Immunoreactivity for the GluN2A subunit (Fig. 5 C2) followed a similar pattern to that of the GluA1 and GluA4 subunits. However, their association with dystrophin-CT immunopositive clusters (Fig. 5 C3-4) appeared less cogent, compared to their AMPA receptor counterparts, with only a minority of overlap being evident, which was borne out by the colocalisation analyses. The MOC for dystrophin-CT signal overlapping with GluN2A was 0.15 ± 0.01, *N* = 5 animals, while the MOC for GluN2A signal overlapping with dystrophin-CT was 0.12 ± 0.01 (Fig. 5 C5). This could be because GluN2A is reportedly located on neighbouring interneurons and not considered to be expressed by PCs themselves (Bidoret et al. 2015). In contrast to all other subunits investigated, immunoreactivity for GluD2 showed the strongest association with signal for dystrophin-CT, with the majority of clusters located in close association, or overlapping with dystrophin (Fig. 5 D1-4). This was confirmed by the colocalisation analyses. The MOC for dystrophin-CT signal overlapping with GluD2 was 0.66 ± 0.01, *N* = 5 animals, while the MOC for GluD2 signal overlapping with dystrophin-CT was 0.37 ± 0.01 (Fig. 5 D5). Collectively, these data suggest that specific glutamate receptor subtypes, GluD2 in particular, could being part of, or directly associated with the DAPC in the cerebellum, and their expression and function could be altered in dystrophinopathy.

### Specific iGluR Subunit Expression is Altered by the Total Dystrophin Absence, but not Dp427 Loss

**Fig. 6:**
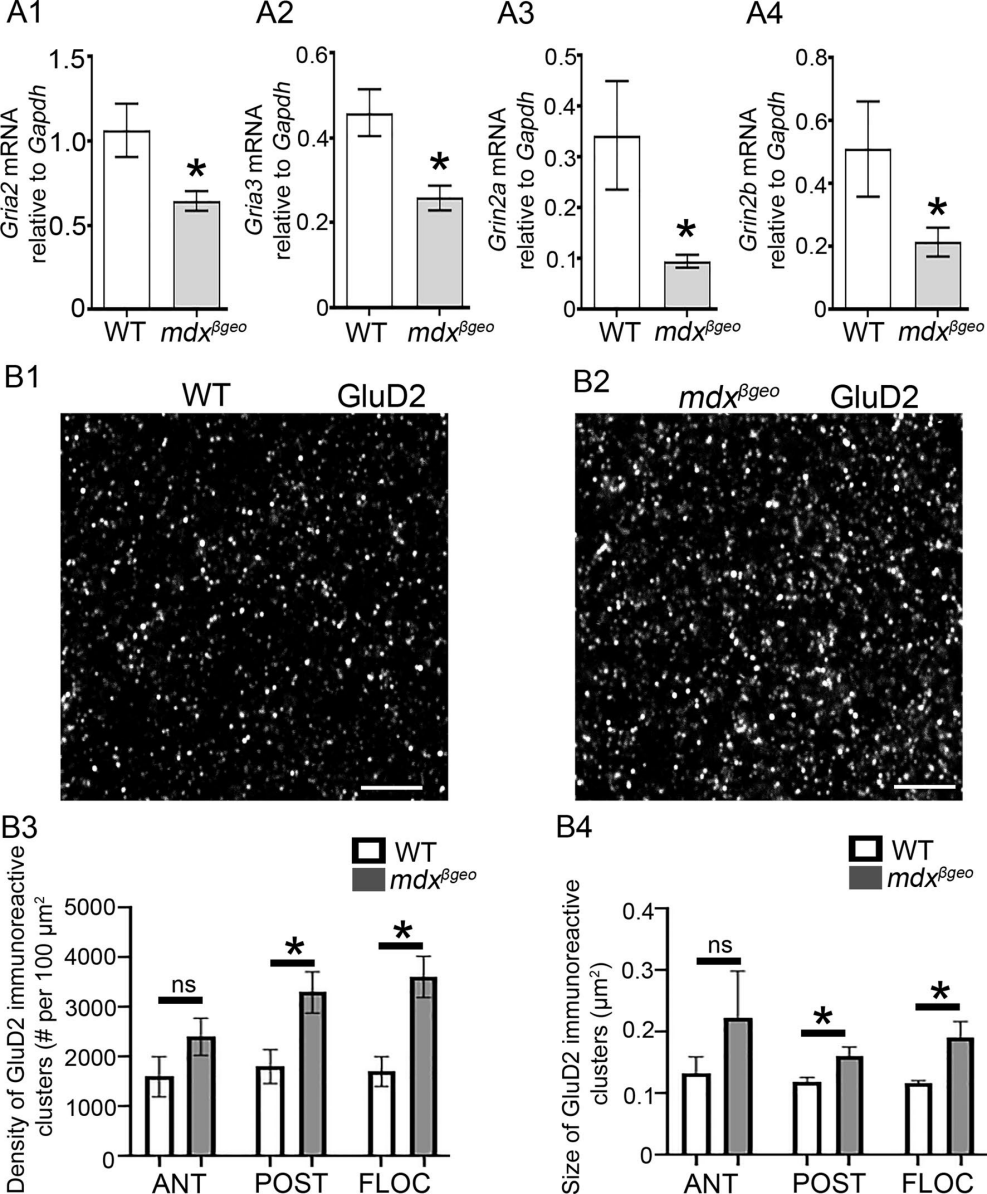
The absence of dystrophin results in changes in the expression of specific iGluR subunits. (A) quantification of the expression levels of iGluR subunit*-*encoding mRNA in homogenates of cerebellar vermis from WT and mdx^βgeo^ mice relative to the *Gapdh* reference gene. The bars represent the means ± SEM. (A1–2) Unpaired Student’s *t *test: * *p* < 0.05; (A3–4), Mann–Whitney *U* test:* *p* < 0.05. (B) Representative confocal micrographs of GluD2 immunofluorescence in the cerebellar molecular layer in tissue from (B1) WT and (B2) mdx^βgeo^ mice, processed under identical conditions. There is a noticeable increase in the size and density of clusters. Quantification of (B3) the density and (B4) size of GluD2-immunopositive clusters. Ant: interior lobe of the cerebellar vermis; Pos: posterior lobe; Floc; flocculonodular lobe. Bars represent (A1–2, B) the mean, and error bars the SEM, and (A3) the median and error bars the interquartile range. * *P* < 0.05 unpaired Student’s *t *test. NS *p* ≥ 0.05. Scale bars: 2 µm

Since we observed immunoreactive puncta for putative-truncated dystrophins in close association with excitatory synapses in the cerebellar molecular layer, we, therefore, assessed whether the expression of the predominant iGluR transcripts was altered in the absence of dystrophin. This analysis was undertaken at the RNA and protein levels in mdx and mdx^βgeo^ cerebellar samples, to compare the full-length vs. total dystrophin absence, respectively. In samples from mdx^βgeo^ mice, qRT-PCR analyses revealed that the relative mRNA levels for the G*ria2* (*P* = 0.0181 unpaired Student’s *t* test,* N* = 12 animals), G*ria3* (*P* = 0.0034, unpaired Student’s *t* test,* N* = 12 animals), G*rin2a* (*P* = 0.0014, *U* = 14, Mann–Whitney *U* test,* N* = 12 animals), and *Grin2b* (*P* = 0.0426, unpaired Mann–Whitney *U* test, *N* = 12 animals) were significantly decreased compared to wild type controls (Fig. 6 A). However, the *Gria1*, *Gria4*, *Grin1,* and *Grid2* transcripts in mdx^βgeo^ cerebella were not statistically significantly altered (SI Table 4). Importantly, we detected no significant differences in the iGluR mRNA expression levels in mdx mice (SI Table 4), further supporting an association of the short-form dystrophins with excitatory synapses. In order to examine whether these dystrophin-related alterations in iGluR subunits were specific to the cerebellum, we also examined the mRNA expression of AMPA and NMDA receptor subunits within the hippocampus, a brain region enriched with iGluRs. We detected no significant differences in the iGluR mRNA expression levels in mdx^βgeo^ hippocampi, compared to the WT mice (SI Table 5).

Since the expression of transcripts encoding select AMPA and NMDA subunits was altered in mdx^βgeo^ mice, we then examined whether this manifested at the protein level, as a change in the distribution or amount of receptor proteins. In order to control for the rostro-caudal distribution of cerebellar circuits, three regions were targeted: the anterior, posterior, and flocculonodular lobes of the cerebellar vermis. Semi-quantitative analysis of GluD2 immunoreactivity revealed a significant increase in both the density (Posterior lobe, *p* = 0.0236; flocculonodular lobe, *p* = 0.006, unpaired Student’s *t* test;* n* = 5 animals), and size of clusters (Posterior lobe, *p* = 0.0423; Flocculonodular lobe, *p* = 0.0271, unpaired Student’s t test;* N* = 5 animals) (Fig. 6 B). However, there were no statistically significant changes in immunoreactivity for the GluA1–4 and GluD1 subunits (SI Table 6). Collectively, these data demonstrate that loss of truncated dystrophins within the cerebellum causes transcriptional downregulation of specific iGluRs, which is mostly compensated at the protein level but, unexpectedly, accompanied by significantly greater GluD2 clusters, which showed the strongest association with dystrophin-CT.

### Putative Truncated Dystrophins are Located Adjacent to Glial P2RX7s in the Cerebellum

**Fig. 7:**
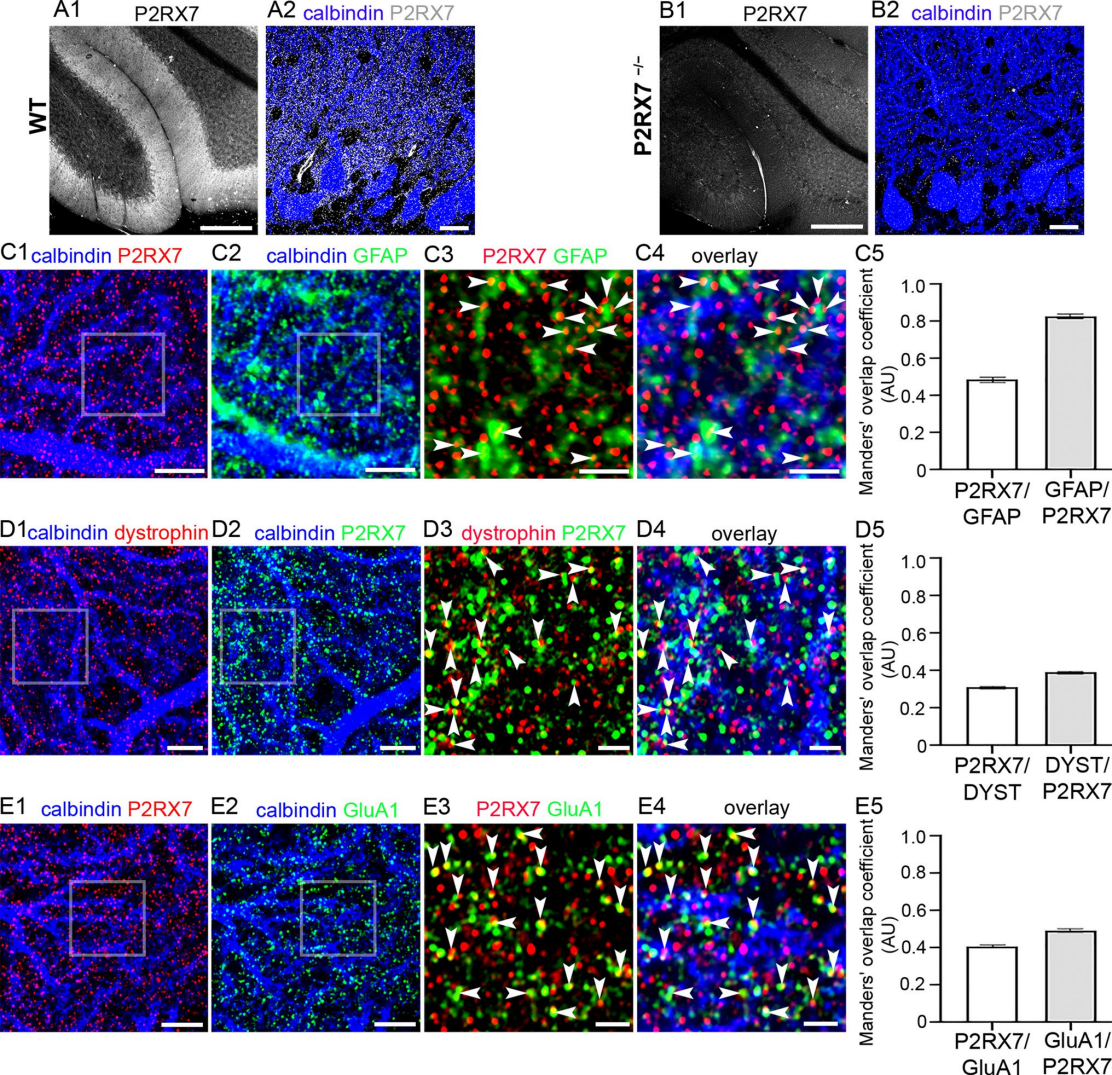
Dystrophin is associated with P2RX7 receptors. (A) immunoreactivity for P2RX7 in the cerebellar cortex is enriched in the molecular layer of WT mice. (B) confirmation of the specificity of the P2RX7 immunoreactivity, with no specific signal detected in tissue from P2RX7 gene-deleted mice. (C1–4) in tissue from a GFP-GFAP mouse, immunoreactivity for P2RX7 presents as individual clusters that frequently overlap (arrowheads) or appose glial processes, identified by GFP signal. (C5) quantification of the relative overlap of P2RX7 signal with GFP-GFAP and GFP-GFAP with P2RX7, according to the Manders’ overlap coefficient method. The bars represent the means and the errors the SEM. *N* = 5 animals. (D1–4) dystrophin-CT immunoreactive clusters are located immediately adjacent to those immunoreactive for P2RX7 (arrowheads). This suggests that P2RX7 is located in glial processes that contact regions of the PC dendrites that express dystrophin. (D5) quantification of the relative overlap of P2RX7 signal with dystrophin-CT and dystrophin-CT with P2RX7, according to the Manders overlap coefficient method. The bars represent the means, and the errors the SEM. *N* = 5 animals. (E1–4) P2RX7 immunoreactive clusters either overlap (arrowheads) or are located adjacent to GluA1 immunopositive clusters. Since GluA1-containing receptors are expressed in both PCs and Bergman glia, P2RX7s could interact with both pre- and postsynaptic GluA1 receptors. (E5) quantification of the relative overlap of P2RX7 signal with GluA1 and GluA1 with P2RX7, according to the Manders’ overlap coefficient method. The bars represent the means, and the errors the SEM. *N* = 5 animals. Scale bars: (**a**, **b**) 200 µm: (**b**, **c**, **d** 1–2) 5 µm; (**b**, **d** 3–4) 2 µm; (**c** 3–4) 1 µm

Ablation of P2RX7 not only alleviated muscle pathology but also results in improvements in cortex-mediated object recognition memory and in reduced anxiety-like behaviour in mdx (Dp427-null) mice (Sinadinos et al. 2015). These data indicate the important role of P2RX7 in the dystrophic brain, but whether this is linked to inflammation (Gorecki 2019; Rae and O'Malley 2016b), or cooperation with GABA and glutamate receptors (Papp et al. 2004) is unknown. Therefore, we explored whether P2RX7s are localised in proximity to dystrophin. Immunoreactivity for P2RX7 was enriched in the molecular layer of the cerebellum, with lower levels of staining evident in the granular cell layer (Fig. 7A), in agreement with previous studies (Atkinson et al. 2004). The specificity of the P2RX7 antibody staining was confirmed in samples from P2RX7 knockout mice, in which no specific signal was detected (Fig. 7B). Previous reports indicate the P2RX7 in the cerebellum to be located in Bergmann glia (Habbas et al. 2011; Kaczmarek-Hajek 2018). We confirmed this in tissue from eGFP-GFAP mice, with P2RX7 immunoreactive clusters overlapping with eGFP-containing processes within the molecular layer, which represents Bergman glia profiles (Fig. 7 C1-4). The MOC for P2RX7 signal overlapping with GFP-GFAP was 0.49 ± 0.01, *N* = 5 animals, while the MOC for GFP-GFAP signal overlapping with P2RX7 was 0.83 ± 0.01 (Fig. 7 C5), confirming previous reports of the significant association of P2RX7 expression with glial elements.

The pattern of P2RX7 immunoreactivity closely resembled that of dystrophin in the cerebellum. We, therefore, explored whether there was any association between these molecules. Within the molecular layer, clusters immunoreactive for dystrophin-CT were in close apposition to those immunopositive for P2RX7, putatively on Bergmann glia processes (Fig. 7 D1-4). The MOC for P2RX7 signal overlapping with dystrophin-CT was 0.31 ± 0.01, *N* = 5 animals, while the MOC for dystrophin-CT signal overlapping with P2RX7 was 0.39 ± 0.01 (Fig. 7 D5). Since Bergman glia processes closely contact PC spines and excitatory synapses, we next explored whether P2RX7 immunolabelling could be associated with signal for iGluRs, in a manner similar to dystrophin. The respective immunoreactivity patterns for P2RX7 and GluA1 closely mirrored one another (Fig. 7 E1-4), with individual clusters either overlapping or being located in close apposition. The MOC for P2RX7 signal overlapping with GluA1 was 0.41 ± 0.01, *N* = 5 animals, while the MOC for GluA1 signal overlapping with P2RX7 was 0.49 ± 0.01 (Fig. 7E5). This suggests that P2RX7 is expressed in Bergmann glia profiles which are most likely adjacent to glutamatergic synapses. No association was observed between P2RX7 and dystrophin on somatic and proximal dendritic compartments corresponding to Dp427, suggesting that it is specifically associated with isoforms located in glutamatergic synapses, but not inhibitory synapses on PCs. Furthermore, P2RX7 located at PC synapses could have implications for neuronal signalling in the dystrophic brain. Indeed, electrophysiological evidence has shown that P2RX7 modulates presynaptic release of glutamate by neurons (Atkinson et al. 2004; Ireland et al. 2004; Leon et al. 2008) and astrocytes (Duan et al. 2003). Thus, the co-location of P2RX7 and truncated dystrophins in excitatory synaptic domains raises the question whether the absence of dystrophin, such as in DMD, impacts on P2RX7 function and, consequently, its contribution to glutamate-mediated functions in the brain, which apart from neurotransmission, also include neuroinflammation (Volpi et al. 2012).

### Loss of all DMD Gene Products Results in Decreased Brain Expression of P2rx7 and Immune Modulators

**Fig. 8:**
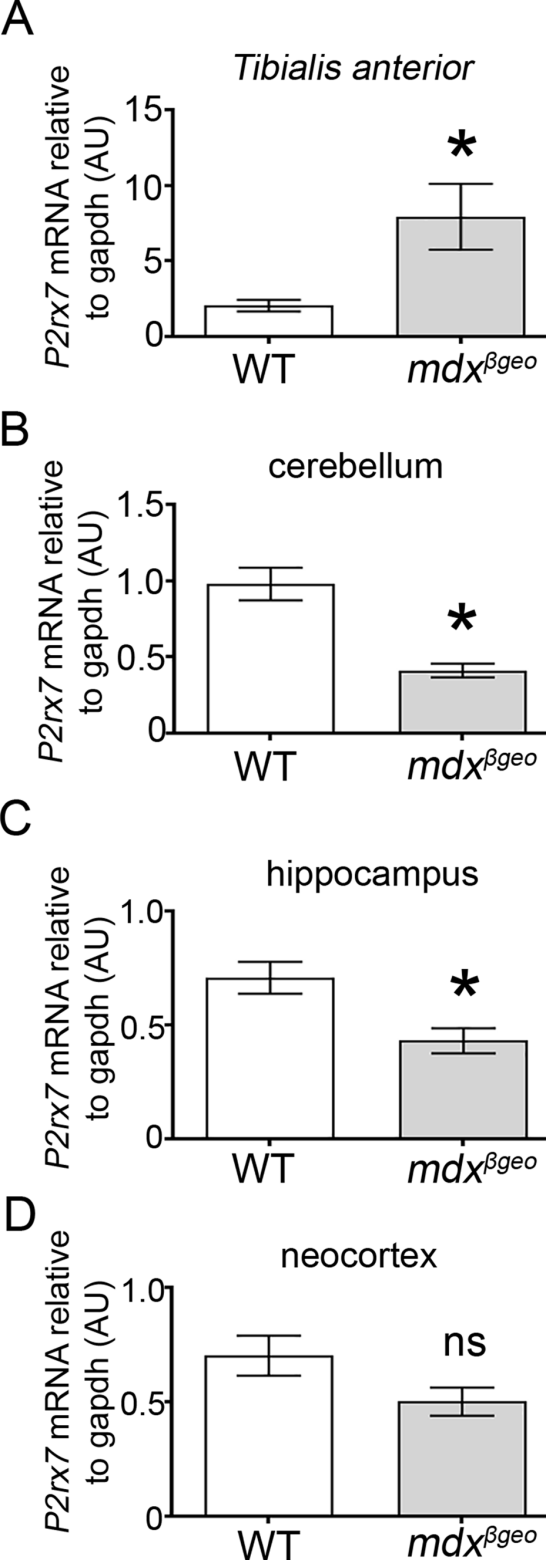
The absence of dystrophin results in opposing changes in *P2rx7* expression levels in muscle and brain. Quantification of P2RX7-encoding mRNA in (A) hind-limb tibialis anterior muscle, (B) cerebellum, (C) hippocampus, and (D) neocortex of WT and mdx^βgeo^ mice. Bars represent the mean and error bars the SEM; * p < 0.05; unpaired Student’s *t* test. *NS* not significant

Previous evidence indicates that the deletion of dystrophin results in an increase in muscle P2RX7 expression, which is understood to be instrumental in driving muscle pathology (Sinadinos et al. 2015). We, therefore, explored whether brain P2RX7 expression is also altered in the absence of dystrophin, and whether any changes in basal immune function could be detected. We first confirmed that, like in mdx, hind-limb *tibialis anterior* (TA) muscle, *P2rx7* expression was also significantly increased in TA samples from mdx^βgeo^ mice (*P* = 0.04; unpaired Student’s t test, *N* = 7 animals) (Fig. 8A). In contrast to muscle, in brain samples from these same animals, qRT-PCR revealed that *P2rx7* expression was significantly decreased in samples from the cerebellum (*p* = 0.0008, unpaired Student’s t test,* N* = 13 animals) (Fig. 8B) and hippocampus (*p* = 0.0049, unpaired Student’s t test, *N* = 12 animals) (Fig. 8C), but not the neocortex (*p* = 0.1086, unpaired Student’s t test,* N* = 4 animals) (Fig. 8D). In contrast, while we confirmed a significant increase in *P2rx7* expression in mdx muscle, (*p* = 0.0281, unpaired Student’s *t* test,* N* = 5 animals), there were no differences in this purinoceptor transcript levels in mdx cerebellar samples (WT: 0.814 ± 0.063 *P2rx7* mRNA level relative to *Gapdh vs* mdx: 0.664 ± 0.052 *P2rx7* mRNA level relative to *Gapdh*; *p* = 0.0801; *N* = 11 animals). These data indicate that the loss of truncated dystrophins alters the transcription of this key inflammatory receptor in a tissue-specific manner.

**Fig. 9:**
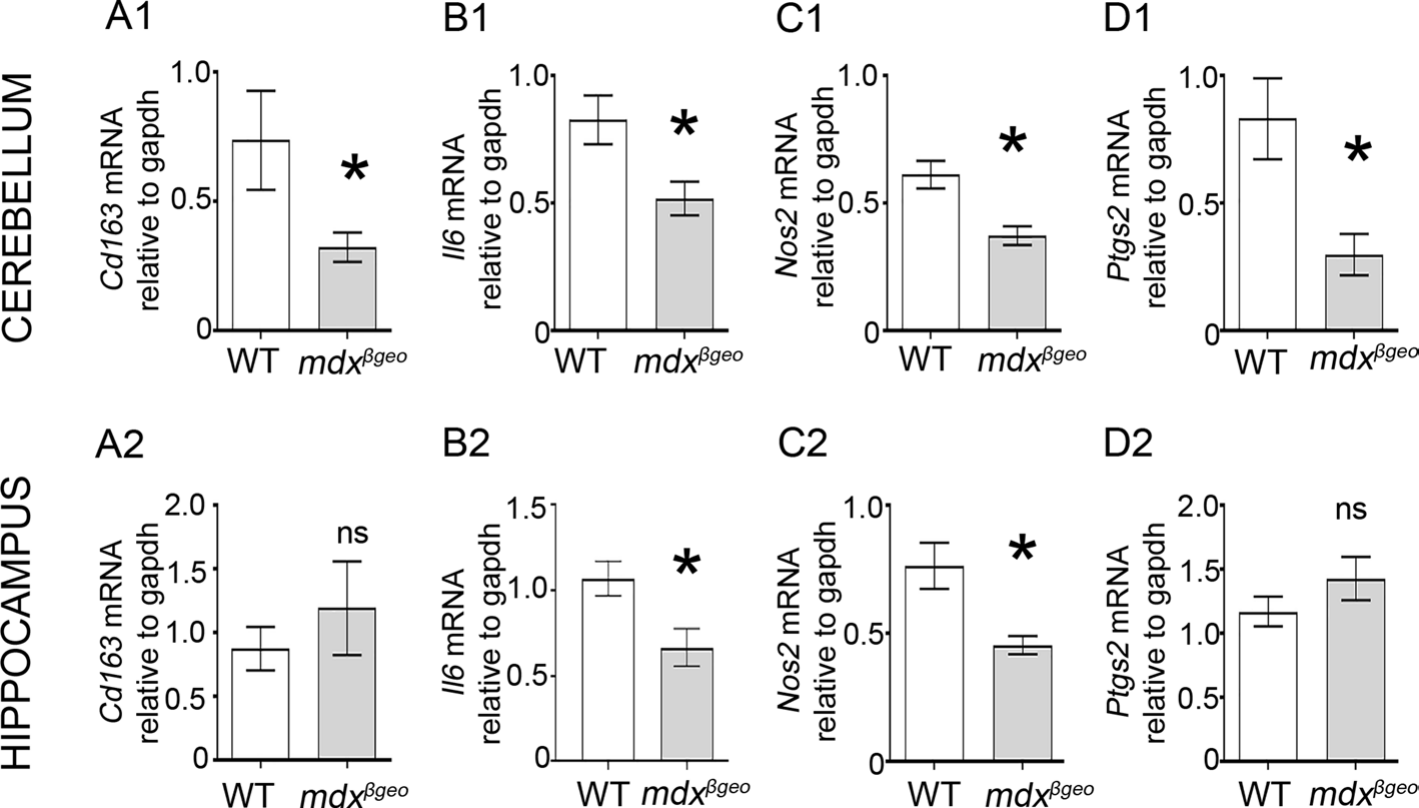
The absence of dystrophin results in a decrease in the basal expression levels of specific immune modulators in the cerebellum and hippocampus. Quantification of pro-inflammatory cytokine-encoding mRNA within the (**a**) cerebellum and (**b**) the hippocampus, in samples from WT and mdx^βgeo^ mice. Bars represent the mean and error bars the SEM* (A, D1, 3–4) Unpaired Student’s *t *test: * *p* < 0.05; (D1), Mann–Whitney *U* test:* *p* < 0.05. *NS* not significant

P2RX7 activation triggers microglial activation and cytokine release (Barbera-Cremades et al. 2012; Di Virgilio et al. 1989; Steinberg and Silverstein 1987), thus, driving inflammatory responses. Therefore, we investigated whether the decreased *P2rx7* mRNA observed in the brain of mdx^βgeo^ mice was indicative of a broader alteration of basal inflammation. qRT-PCR analysis revealed decreased expression of mRNA for *Cd163* (*P* = 0.0364, unpaired Student’s t test;* N* = 10 animals) (Fig. 9 A1), *Il6* (*P* = 0.0248, unpaired Student’s t test;* N* = 6 animals) (Fig. 9 B1), *Nos2* (*P* = 0.0019, unpaired Student’s t test;* N* = 12 animals) (Fig. 9 C1) and *Ptgs2* (*P* = 0.0032, *U* = 17, Mann–Whitney *U* test;* N* = 11 animals) (Fig. 9 D1). In contrast, in the hippocampus, there were similar decreases in expression of *Il6* (*P* = 0.0297, unpaired Student’s t test;* N* = 6 animals) (Fig. 9 B2) and *Nos2* (*P* = 0.0051, unpaired Student’s t test;* N* = 8 animals) (Fig. 9 C2), and there were no significant changes in expression of *cd163* (*P* = 0.4295, unpaired Student’s t test;* N* = 8 animals) (Fig. 9 A2) and *Ptgs2* (*P* = 0.2459, unpaired Student’s t test; *N* = 8 animals) (Fig. 9 D2). Collectively, these data demonstrate that in the absence of brain dystrophins, there are changes in the intrinsic expression of key immune mediators in a region-specific manner.

## Discussion

The data demonstrate that PCs target different DMD gene products to molecularly and functionally distinct populations of synapses. This suggests that an individual cell type, the PC, exploits different molecular variants of the dystrophin protein to process diverse forms of information transferred at excitatory and inhibitory synapses. Furthermore, the data reveal that in the cerebellum, truncated DMD gene products are located in close contact with P2RX7s, which are known to mediate the inflammatory component of muscle pathology in DMD, as well as being associated with cognitive performance in dystrophic mice. However, in contrast to their increased expression in dystrophic muscle, expression of P2RX7, alongside the levels of specific pro-inflammatory immune mediators, is decreased in the brain of mice lacking all dystrophins. This suggests that the immune component of DMD pathology in the brain could be distinct to that in other organs. This is important because the maintenance of homeostatic levels of pro- and anti-inflammatory mediators is required in facilitating a rapid, transient inflammatory response in the presence of invading pathogens or sterile trauma, and imbalances in these mediators can potentially render the brain unable to adequately react to such threats. Additionally, homeostatic levels of immune molecules are critical to normal development and function of neuronal circuits and synaptic plasticity, and therefore, alterations in these molecules may represent a mechanism underlying developmental cognitive impairment that occurs in DMD due to the absence of dystrophin.

### The Expression of Dystrophin in the Cerebellar Cortex is Gene Product and Synapse Specific

The identification of the precise subcellular expression location of truncated dystrophins, at the protein level, has remained elusive. Previously published immunoblot data, using the same dystrophin-CT antibody used in this study, suggest that the predominant *truncated* dystrophins expressed in the adult rodent cerebellum are most likely Dp71 and Dp140 (Hendriksen et al. 2016). Dystrophin immunoreactivity for putative isoforms was concentrated on dendritic spines on the distal dendrites of PCs throughout the molecular layer, which is the domain of excitatory transmission via climbing and parallel fibre inputs. This builds on functional evidence using mouse models of short dystrophin isoforms, such as Dp71-null mice which exhibit altered synaptic input and plasticity at climbing fibre-PC synapses (Helleringer et al. 2018). These data broaden our understanding of the role of truncated dystrophins in cerebellar function in light of the known association between these afferent systems and cerebellar plasticity (Kawato et al. 2020), and the association between dystrophin deficiency and cerebellum-associated cognitive processes (Helleringer et al. 2018; Vicari et al. 2018). However, an important caveat is that in contrast to mice, in the human brain, DMD expression is lowest in the cerebellum (Doorenweerd et al. 2017).

The selective targeting of putative-truncated dystrophin to excitatory synapses appears to hold true beyond the cerebellum as well. Indeed, within the hippocampus, co-immunoprecipitation analyses suggests Dp71 clustering with iGluR subunits and PSD-95 in rat, which is accompanied by altered plasticity and pre- and postsynaptic ultrastructure in Dp71-null mouse CA1 pyramidal neurons (Daoud et al. 2008; Miranda et al. 2011; Miranda et al. 2009). This raises the question of the precise functional role of Dp71 within glutamatergic synapses. Since the full-length Dp427 variant is known to anchor α1 and α2 subunit-containing GABA_A_R (Brunig et al. 2002; Knuesel et al. 1999), it is reasonable to speculate that the targeting of Dp71 to glutamatergic synapses sub-serves a similar anchoring role for one or more specific iGluR subunits, especially given previous immunoprecipitation evidence of the association of Dp71 with PSD-95, GluA1–3, and GluN2A–B in rat hippocampal homogenates (Daoud et al. 2008). Indeed, the absence of Dp71 is reported to alter the size and distribution PSD-95 immunoreactive clusters (Helleringer et al. 2018). Since findings of this study indicate that cerebellar expression of the GluA2–3, and GluN2A–B subunits are downregulated in the absence of all dystrophins, it is possible that the loss of Dp71 impairs the ability of PSD-95 to cluster at the synapse, which in turn limits its capacity to anchor specific glutamate receptor subunits at such locations. A possible mechanism for Dp71-DAPC associating with excitatory synapses could be via direct interaction with PSD-95. Indeed, syntrophin, a constituent of the DAPC, contains the PDZ domain, which may facilitate binding with PDZ-binding domains present on iGluR subunits (Traynelis 2010). Thus, the possibility exists that the DAPC is directly associated with iGluRs at the PSD, and that the loss of dystrophin impairs their synaptic anchoring.

This role of truncated dystrophins is supported by a novel finding in this study that dystrophin-null but not the absence of the full-length variant, alters the expression of transcripts encoding for specific AMPA and NMDA receptor subunits. Interestingly, these transcriptional alterations were largely compensated at the protein level, and no changes were detected in the hippocampus. Why dystrophin would impact on the gene expression of these receptors in one brain region and not another is not immediately clear. It should be noted that at least ten Dp71 splice variants has been demonstrated in brain, including the smallest Dp40 dystrophin which has been detected in synapses (Austin et al. 1995). Thus, such differences in cellular or region influences could be linked to the expression of distinct variants. Unfortunately, no available antibodies can discriminate between such splice variants. Such regional differences could also be due to the different contributions of the cerebellum and hippocampus to overall brain function, as well as the functionally distinct principle cells within these structures, these being the GABAergic PCs of the cerebellum, and the glutamatergic pyramidal neurons of the hippocampus. Given the purported roles of dystrophin in anchoring neurotransmitter receptors and thereby influencing synaptic transmission, plasticity of dystrophin isoforms could be distinct, and according to specific functional roles within individual neural circuits, rather than sub-serving a common, brain-wide function. This might account for the diversity of neuropsychiatric symptoms arising from dystrophinopathy.

A notable discovery was the increased level of GluD2 immunoreactivity, suggesting increased expression at the protein level. This is intriguing because it was the one receptor subunit investigated which did exhibit a considerable degree of colocalisation with dystrophin. Simplistically, this could indicate a protein–protein interaction akin to those occurring within the DAPC. This might provide an explanation for the increased expression of GluD2 in the mdx-beta geo mice since this might be required to compensate for the total absence of all dystrophins within this model. The GluD2 receptor is an intriguing element of excitatory cerebellar circuitry owing to both its peculiarity and pivotal role in facilitating cerebellar development and function. Indeed, several knockout studies have demonstrated that GluD2 is essential for circuitry and synaptic organisation and function in the cerebellum (Hashimoto et al., 2009; Ichikawa et al., 2002). Furthermore, the GluD2 subunit is also crucial for the organisation of molecular layer synapses during postnatal development, including PF-PC synapse morphology, and the expression of AMPA receptors (Kakegawa 2008; Uemura 2007; Watanabe and Kano 2011). This is of particular relevance to DMD because the early-juvenile onset and non-progressive nature of neuropsychiatric symptoms point to DMD-dependent cognitive deficits being of developmental origin. Such a developmental deficit may be a consequence of aberrations in the neural progenitor cells development. The expression of PC-specific Dp427 is detectable at 12.5–13 postnatal days of mouse development (Houzelstein et al. 1992), and PC is reported to arise between days 11 and 13 of mouse embryogenesis (Miale and Sidman 1961). Moreover, the protracted postnatal development profile of the cerebellum raises the question whether cerebellar dysfunctions contribute to cognitive impairment originating prenatally. Importantly, GluD2 immunoreactive puncta were larger and more abundant in mdx^βgeo^ mice, and previous evidence shows that Dp71-null mice exhibit impaired long–term potentiation. Future ultrastructural studies on the organisation and development of the mdx^βgeo^ mouse cerebellum will be integral in determining whether the Dp71 isoform contributes to postnatal cerebellar development, and its absence in turn is a contributor to DMD brain pathology.

### Dystrophin and the Basal Immune State of the Brain

Another intriguing finding was the inter-relation between the expression of dystrophin, P2RX7, and cytokines within the cerebellum. Immunohistochemistry revealed the location of P2RX7 at glutamatergic synapses in the cerebellar molecular layer, closely associated with dystrophin. Moreover, P2RX7 mRNA expression in dystrophin-null brains was downregulated. In muscle, dystrophinopathy coincides with a significant P2RX7 upregulation and altered receptor signalling in muscle cells (Young et al. 2015). Furthermore, dystrophic damage triggering a massive release of ATP coincides with the loss of DAPC, with one of its constituents, α-sarcoglycan, being an ecto-ATPase. Increased release combined with reduced ATPase degradation drastically elevate extracellular ATP (eATP) levels. Such high eATP activates P2RX7, which triggers chronic inflammatory and immune responses that exacerbate the dystrophic pathology (Gorecki 2019). In the brain, when activated by high eATP concentrations, P2RX7 also contribute to pro-inflammatory processes by triggering microglial activation and cytokine release (Iwata, 2016; Shieh et al. 2014; Solle et al. 2001), thus, promoting local inflammation.

The loss of dystrophin in the brain and its potential impact on P2RX7 expression and purinergic signalling could influence downstream immune and inflammatory pathways. The current study revealed that, in a stark contrast to its upregulation in dystrophic muscle, the absence of dystrophins results in a loco-specific decrease in brain expression of P2RX7. One explanation would be the different dystrophin isoforms being expressed in muscle and brain. The current data, using the mdx mouse, indicate that the loss of full-length dystrophins does not affect brain P2RX7 expression nor inflammatory mediators. Thus, the loss of only long, or all dystrophins causes P2RX7 upregulation in muscle, the loss of long isoforms in the presence of truncated variants in brain has no effect, while the loss of all isoforms causes P2RX7 downregulation in the cerebellum but not in muscle (our data). This, therefore, suggests that it is rather not the difference in isoform expression but that different dystrophins have different functions in these two organs and, thus, can mediate specific aspects of DMD pathology.

Another possible explanation for the differences in in P2RX7 expression, in either muscle or brain, might be resulting not from its dysregulation due to loss of dystrophins but from the compensatory responses in dystrophic cells. P2RX7 expression allows better adaptability to unfavourable metabolic conditions (Amoroso et al. 2012). Thus, the P2RX7 upregulation in dystrophic muscle cells might be an attempt to reprogram cell metabolism to meet the needs imposed by intrinsic metabolic abnormalities observed in DMD (Sharma et al. 2003) and/or adverse dystrophic muscle environment (reactive oxygen species, inflammation). However, this compensatory P2RX7 upregulation coincides with the aforementioned loss of α-sarcoglycan, which otherwise would eliminate excessive eATP (Sandona and Betto 2009). Therefore, instead of metabolic augmentation, high P2RX7 combined with eATP-rich environment further damage dystrophic muscle (Gorecki 2019). In healthy brain, the eATP concentration is very low (Falzoni et al. 2013). Therefore, brain P2RX7 activation only occurs in pathology, where there is a rise of eATP levels (Bhattacharya and Biber 2016). The increased expression of P2RX7 triggers microglial activation and is invariably associated with neuroinflammation (Monif et al. 2009). Given the already permeable dystrophic blood brain barrier (BBB) (Lien et al. 2012), the inflammatory mediators released from inflamed muscle into the bloodstream could induce insults in brain cells and exacerbate abnormalities caused by the intrinsic dysregulation of neuronal receptor anchorage and signalling. Indeed, the maintenance of homeostatic levels of inflammatory mediators is critical to normal development and function of neuronal circuits and synaptic plasticity. Therefore, it may represent a mechanism contributing to the developmental cognitive impairment in DMD. In this respect, our finding of altered expression of IL-6 (Fig. 9B) is directly relevant as both IL-6 overexpression and gene knockout have been associated with cognitive impairment in mice (Heyser et al. 1997, Burton and Johnson 2012; Donegan et al. 2014). To counter such an inflammatory insult, dystrophic brain cells appear to respond with the reduced expression of P2RX7, which is followed by and/or coincides with the reduced expression of several inflammatory mediators (this work).

An additional consideration is the BBB that permeability increases with the progressive loss of dystrophins (Lien et al. 2012) and can vary across different brain regions (Moinuddin et al. 2000). This could perhaps explain the P2RX7 downregulation observed in dystrophin null but not mdx brains and also in cerebella and hippocampi, but not cortical regions (this data). Furthermore, variability of brain inflammation due to other factors affecting the inflammatory mediators load and the BBB permeability could contribute to the spectrum of brain symptoms in patients with the same DMD mutation. This hypothesis could also explain why ablation of P2RX7 alleviated both muscle and brain phenotypes and improve certain aspects of neocortical-dependent cognition, in the DMD mouse (Sinadinos et al. 2015). P2RX7 ablation reduced muscle inflammation and also the myoblast loss, thus, improving regeneration, while P2RX7 absence in the brain dampened the inflammatory responses, thus, improving certain aspects of cognition.

Collectively, these findings suggest that different dystrophin isoforms are targeted to distinct neural circuits and may mediate specific aspects of the of DMD pathology. This could have important implications for the design of targeted future therapeutic interventions which may need to be isoform specific, in order to address the full spectrum of patient symptoms. It also raises the possibility that underfunctioning of the immune system is a component of DMD brain pathology, particularly those associated with neuro-developmental processes.

## Supplementary Information

Below is the link to the electronic supplementary material.

Supplementary file1 (DOCX 53 KB)

## Data Availability

All data will be deposited and made publicly available in the University of Portsmouth Research Portal (Pure): https://researchportal.port.ac.uk/portal/en/
