# Supplementary material for: Specific Dystrophins Selectively Associate with Inhibitory and Excitatory Synapses of the Mouse Cerebellum and their Loss Alters Expression of P2X7 Purinoceptors and Pro-Inflammatory Mediators
Source: Cell Mol Neurobiol. 2021 Jun 8;42(7):2357–77. doi: 10.1007/s10571-021-01110-6 (PMC9418305; doi:10.1007/s10571-021-01110-6)
Supplement: Supplementary file 1 — Supplementary file1 (DOCX 53 KB) [file 10571_2021_1110_MOESM1_ESM.docx]

**Supplementary Information**

Specific dystrophins selectively associate with inhibitory and excitatory synapses of the mouse cerebellum and their loss alters expression of P2X7 purinoceptors and pro-inflammatory mediators

Torquil Jackson, Mohsen Seifi, Dariusz C Gorecki, Jerome D. Swinny

Table 1

Details of primary antibodies used in the study

| Target | Species | Dilution Factor | Supplier  (Product code) | Specificity/Reference |
| --- | --- | --- | --- | --- |
| Calbindin | Ms | 1:4000 | Swant (#300) | (Celio *et al*., 1990) |
| Calbindin D28k | Go | 1:2000 | Frontier Institute  (Calbindin-Gt-Af310) | (Nakagawa *et al*., 1998) |
| Calbindin D28k | Rb | 1:1000 | Swant | (Celio *et al*., 1990) |
| Dystrophin  (C-Terminus) | Rb | 1:1000 | Abcam (ab15277) | Absence of specific signal in dystrophin-null tissue (This study), (Panzanelli, Früh, & Fritschy, 2017) |
| Dystrophin  (Rod domain) | Ms | 1:50 | Novacastra (DYS-1) | (Knuesel *et al*., 1999) |
| GFAP | Ck | 1:3000 | Millipore (AB5541) | Labelling consistent with previous reports and GFAP-eGFP reporter mouse fluorescence (Jukkola *et al*., 2013; Nolte *et al*., 2001) |
| GluA1 | GP | 1:1000 | Frontier Institute (GluA1-Gp-Af380) | (Fukaya *et al*., 2006; Shimuta *et al*., 2001) |
| GluA1 | Rb | 1:3000 | Frontier Institute (GluA1-Rb-Af690) | (Fukaya *et al*., 2006; Shimuta *et al*., 2001) |
| GluA2 | Rb | 1:2000 | Frontier Institute (GluR2C-Rb-Af1050) | (Fukaya *et al*., 2006; Shimuta *et al*., 2001) |
| GluA3 | Rb | 1:3000 | Frontier Institute (GluR3C-Rb-Af1090) | (Nagy *et al*., 2004) |
| GluA4 | GP | 1:1000 | Frontier Institute (GluA4N-GP-Af640) | (Nagy *et al*., 2004) |
| GluA4 | Rb | 1:1000 | Frontier Institute (GluR4C-Rb-Af160) | (Nagy *et al.,*. 2004) |
| GluD1 | Rb | 1:1000 | Frontier Institute (GluD1C-Rb-Af1390) | (Konno *et al*., 2014) |
| GluD2 | Rb | 1:3000 | Frontier Institute (GluD2C (897-934) –GP-Af1090) | (Konno *et al*., 2014) |
| GluN2A | Rb | 1:1000 | Frontier Institute (GluRe1C-Rb-Af542) | (Watanabe *et al*., 1998) |
| P2X7R | Ms | 1:7500 | Gift from Prof. Francesco DiVirgilio | Absence of specific signal in *P2rx7*-KO mouse tissue (this study). |
| PSD-95 | Gp | 1:1000 | Frontier Institute (PSD95-GP-Af660) | Fukaya and Watanabe, 2000) |
| SAP 97 | Sh | 1:100 | University of Dundee MRC PPU (S552B) | (Sabio *et al*., 2005) |
| VGAT | Go | 1:3000 | Frontier Institute (VGAT-Gt-Af620) | (Miura *et al*., 2006) |
| VGluT1 | Go | 1:5000 | Frontier Institute (VGluT1-GP-Af310) | (Miyazaki *et al*., 2003) |
| VGluT1 | GP | 1:3000 | Frontier Institute (VGluT1-GP-Af570) | (Miyazaki *et al*., 2003) |
| VGluT2 | GP | 1:3000 | Frontier Institute (VGluT2-GP-Af810) | (Miyazaki *et al*., 2003) |
| VGluT2 | Rb | 1:2000 | Synaptic Systems (135403) | (Sperk *et al*., 2003) |

Table 2

Colocalisation analyses using the Manders’ overlap coefficient (MOC) method.

Data are presented as the mean ± SEM, N = 5 WT animals

|  | **Dystrophin MOC** | **Marker MOC** | **% of dystrophin MOC after mirroring dystrophin image** |
| --- | --- | --- | --- |
| Dystrophin & VGAT | 0.098 ± 0.02 | 0.223 ± 0.06 | 15 ± 4 |
| Dystrophin & PSD-95 | 0.374 ± 0.02 | 0.318 ± 0.01 | 8 ± 1 |
| Dystrophin & SAP97 | 0.338 ± 0.01 | 0.442 ± 0.01 | 9 ± 3 |
| Dystrophin & VGLUT1 | 0.176 ± 0.01 | 0.110 ± 0.02 | 13 ± 6 |
| Dystrophin & VGLUT2 | 0.111 ± 0.03 | 0.134 ± 0.01 | 14 ± 4 |
| Dystrophin & GluA1 | 0.312 ± 0.01 | 0.214 ± 0.02 | 9 ± 2 |
| Dystrophin & GluA4 | 0.314 ± 0.02 | 0.202 ± 0.01 | 11 ± 3 |
| Dystrophin & GluN2a | 0.156 ± 0.01 | 0.120 ± 0.01 | 12 ± 3 |
| Dystrophin & GluD2 | 0.656 ± 0.01 | 0.374 ± 0.01 | 3 ± 1 |
|  |  |  |  |
|  | **P2RX7 MOC** | **Marker MOC** | **% of P2RX7 MOC after mirroring P2RX7 image** |
| P2RX7 & GFAP | 0.486 ± 0.01 | 0.828 ± 0.01 | 4 ± 1 |
| P2RX7 & dystrophin | 0.310 ± 0.01 | 0.390 ± 0.01 | 8 ± 2 |
| P2RX7 & GluA1 | 0.410 ± 0.01 | 0.494 ± 0.01 | 5 ± 1 |

Table 3. TaqMan® probes used for qRT-PCR gene expression assays in this study

| **Mouse Gene** | **Encoding Protein** | **Reference (Life Technologies)** |
| --- | --- | --- |
| *Cd163* | CD163 | Mm00474091_m1 |
| *Gapdh* | GAPDH | [Mm99999915_g1](https://www.thermofisher.com/taqman-gene-expression/product/Mm99999915_g1?CID=&ICID=&subtype=) |
| *Gria1* | GluA1 | [Mm00433753_m1](https://www.thermofisher.com/taqman-gene-expression/product/Mm00433753_m1?CID=&ICID=&subtype=) |
| *Gria2* | GluA2 | Mm00442822_m1 |
| *Gria3* | GluA3 | [Mm00497506_m1](https://www.thermofisher.com/taqman-gene-expression/product/Mm00497506_m1?CID=&ICID=&subtype=) |
| *Gria4* | GluA4 | [Mm00444754_m1](https://www.thermofisher.com/taqman-gene-expression/product/Mm00444754_m1?CID=&ICID=&subtype=) |
| *Grid2* | GluD2 | [Mm00492353_m1](https://www.thermofisher.com/taqman-gene-expression/product/Mm00492353_m1?CID=&ICID=&subtype=) |
| *Grin1* | GluN1 | [Mm00433790_m1](https://www.thermofisher.com/taqman-gene-expression/product/Mm00433790_m1?CID=&ICID=&subtype=) |
| *Grin2a* | GluN2A | [Mm00433802_m1](https://www.thermofisher.com/taqman-gene-expression/product/Mm00433802_m1?CID=&ICID=&subtype=) |
| *Grin2b* | GluN2B | [Mm00433820_m1](https://www.thermofisher.com/taqman-gene-expression/product/Mm00433820_m1?CID=&ICID=&subtype=) |
| *Il6* | Interleukin 6 | [Mm00446190_m1](https://www.thermofisher.com/taqman-gene-expression/product/Mm00446190_m1?CID=&ICID=&subtype=) |
| *Nos2* | Inducible NOS | Mm00440502_m1 |
| *P2rx7* | P2X7R | Mm01199500_m1 |
| *Ptgs2* | COX-2 | Mm00478374_m1 |

| **Subunit gene (encoded protein)** | **Mean ± SEM (median)** | | **P** | **N** |  | **Mean ± SEM (median)** | | **P** | **N** |
| --- | --- | --- | --- | --- | --- | --- | --- | --- | --- |
|  | **C57 BL/10** | **mdx** |  |  |  | **C57 BL/6** | **β-geo** |  |  |
| **AMPA-type Subunits** | | | | | | | | | |
| **Gria1 (GluA1)** | 0.7880 ± 0.0512 | 0.753 ± 0.0490 | 0.6364 | 5, 5 |  | 0.586 ± 0.0556 | 0.665 ± 0.0465 | 0.2848 | 11, 12 |
| **Gria2 (GluA2)** | 0.8846 ± 0.0980 | 0.885 ± 0.1296 | 0.9997 | 10, 11 |  | 1.063 ± 0.1590 | 0.644 ± 0.0576 | 0.0181; ***** | 11, 12 |
| **Gria3 (GluA3)** | 0.8150 ± 0.0606 | 0.771 ± 0.0491 | 0.5747 | 10, 11 |  | 0.459 ± 0.0548 | 0.259 ± 0.0292 | 0.0034; ****** | 11, 12 |
| **Gria4 (GluA4)** | 0.5852 ± 0.0686 | 0.635 ± 0.0398 | 0.4902 | 10, 10 |  | 1.474 ± 0.167 | 1.178 ± 0.159 | 0.213 | 11, 12 |
| **NMDA-type Subunits** | | | | | | | | | |
| **Grin1 (GluN1)** | † (1.1520) | † (0.9410) | 0.1111 (3) | 5, 4 |  | † (0.5315) | † (0.4620) | 02522 (42) | 10, 12 |
| **Grin2a (GluN2A)** | † (0.7100) | † (0.8010) | 0.0943 (24) | 10, 9 |  | † (0.1560) | † (0.0930) | 0.0014; ****** (14) | 10, 12 |
| **Grin2b (GluN2B)** | † (0.8280) | † (0.6940) | 0.2761 (45) | 10, 10 |  | † (0.3730) | † (0.1495) | 0.0426; ***** (29) | 10, 12 |
| **Delta-type Subunits** | | | | | | | | | |
| **Grin2d (GluD2)** | † (0.9975) | (1.076) | 0.6433 (48) | 10, 11 |  | † (0.6380) | † (0.5965) | 0.5265 (22) | 7, 8 |

Table 4

Summary of relative levels of iGluR subunit-encoding mRNAs in the cerebellum of mdx, mdx-β-geo, and WT mice.

Numerical values denote levels of detected mRNA relative to *Gapdh*. *, *P <* 0.05; **, *P <* 0.01, Student’s *t-*test or Mann-Whitney *U* test.; 1*N* = one animal. †: nonparametric sample distribution—Mann-Whitney *U* test used.

| **Subunit gene (encoded protein)** | **Mean ± SEM (median)** | | **P (U)** | **N** |  | | **Mean ± SEM** | | **P** | **N** |  |
| --- | --- | --- | --- | --- | --- | --- | --- | --- | --- | --- | --- |
|  | **C57 BL/6** | **β-geo** |  |  |  | | **C57 BL/6** | **β-geo** |  |  |  |
| **AMPA-type Subunits** | | | | | **NMDA-type Subunits** | | | | | | |
| **Gria1 (GluA1)** | 0.7031 ± 0.0602 | 0.8610 ± 0.0717 | 0.1292 | 11, 12 | **GluN1** | 0.5233 ± 0.0639 | | 0.5838 ± 0.1187 | 0.6662 | 11, 12 |  |
| **Gria2 (GluA2)** | † (1.377) | † (1.266) | 0.6194  (52) | 10, 11 | **GluN2A** | 0.7034 ± 0.1519 | | 0.8163 ± 0.1603 | 0.6159 | 11, 12 |  |
| **Gria3 (GluA3)** | 0.8150 ± 0.0606 | 0.771 ± 0.0491 | 0.5747 | 10, 11 | **GluN2B** | 0.8905 ± 0.1713 | | 0.7255 ± 0.1410 | 0.4624 | 11, 12 |  |

Table 5

Summary of relative levels of iGluR subunit-encoding mRNAs in the hippocampus of mdx^β-geo^, and WT mice.

Numerical values denote levels of detected mRNA relative to *Gapdh*. *, *P* < 0.05; **, *P* < 0.01, Student’s *t*-test or Mann-Whitney *U* test.; 1N = one animal. †: nonparametric sample distribution—Mann-Whitney *U* test used.

Table 6

Summary of quantitative cluster analysis of AMPA- and Delta-type glutamate receptor subunits in the cerebellum of WT and mdx-β-geo mice. The density and area covered by immunopositive clusters were analysed. Ant: Anterior lobe of the cerebellar vermis; Pos: Posterior lobe; Floc; flocculonodular lobe. Five fields of view were captured from one specimen from each animal and numerical data averaged to create one data point. Mean ± SEM. Student’s *t*-test: *, *P* < 0.05; **, *P* < 0.01; *N* = 5 animals

|  | **Lobe** | **Cluster Density per 100 µm^2^** | | |  | **Cluster Size (µm^2^)** | | |
| --- | --- | --- | --- | --- | --- | --- | --- | --- |
|  |  | **Mean ± SEM** | | |  | **Mean ± SEM** | | |
|  |  | **WT** | **β-geo** | ***P*** |  | **WT** | **β-geo** | ***P*** |
| **AMPA-type Subunits** | | | | | | | | |
| **GluA1** | **Ant** | 2269.2 ± 290.08 | 2019.6 ± 573.2 | 0.4966 |  | 0.1818 ± 0.03 | 0.2434 ± 0.08 | 0.4966 |
|  | **Pos** | 2070.8 ± 191.04 | 2158.0 ± 186.6 | 0.7519 |  | 0.2012 ± 0.02 | 0.1786 ± 0.01 | 0.3638 |
|  | **Floc** | 1903.6 ± 107.52 | 2198.8 ± 229.04 | 0.2768 |  | 0.184 ± 0.01 | 0.194 ± 0.001 | 0.5232 |
| **GluA2** | **Ant** | 4020 ± 274.56 | 3118.8 ± 666 | 0.2941 |  | 0.1564 ± 0.01 | 0.1636 ± 0.01 | 0.762 |
|  | **Pos** | 2382.8 ± 276.72 | 2173.6 ± 133.84 | 0.4892 |  | 0.1395 ± 0.01 | 0.1397 ± 0.01 | 0.9806 |
|  | **Floc** | 2698 ± 424 | 1970 ± 587.2 | 0.3718 |  | 0.1598 ± 0.02 | 0.1448 ± 0.02 | 0.648 |
| **GluA3** | **Ant** | 1999.2 ± 260.56 | 2626 ± 107.44 | 0.0568 |  | 0.1892 ± 0.02 | 0.2116 ± 0.01 | 0.3047 |
|  | **Pos** | 992.8 ± 225.36 | 1588 ± 262.04 | 0.1234 |  | 0.1174 ± 0.01 | 0.1259 ± 0.008 | 0.4888 |
|  | **Floc** | 985.2 ± 361.68 | 1236 ± 151.4 | 0.5403 |  | 0.1302 ± 0.01 | 0.1248 ± 0.008 | 0.1248 |
|  | **Lobe** | **Cluster Density per 100 µm^2^** | | |  | **Cluster Size (µm^2^)** | | |
|  |  | **Mean ± SEM** | | |  | **Mean ± SEM** | | |
|  |  | **WT** | **β-geo** | **P** |  | **WT** | **β-geo** | ***P*** |
| **GluA4** | **Ant** | 3099.6 ± 513.6 | 3329.6 ± 419.2 | 0.7375 |  | 0.1747 ± 0.03 | 0.1591 ± 0.01 | 0.658 |
|  | **Pos** | 2833.6 ± 458 | 3248.4 ± 533.6 | 0.5715 |  | 0.169 ± 0.03 | 0.1674 ± 0.02 | 0.9631 |
|  | **Floc** | 2808 ± 148.84 | 3279.6 ± 486.8 | 0.3815 |  | 0.1504 ± 0.01 | 0.1755 ± 0.02 | 0.3378 |
| **Delta-type Subunits** | | | | | | | | |
| **GluD1** | **Ant** | 1602.9 ± 408.6 | 2407.9 ± 374.0 | 0.1841 |  | 0.14056 ± 0.014 | 0.1828 ± 0.01 | 0.0974 |
|  | **Pos** | 693.4 ± 101.0 | 612.7± 124.4 | 0.6398 |  | 0.1388 ± 0.006 | 0.12524 ± 0.004 | 0.0871 |
|  | **Floc** | 776.6 ± 152.9 | 1104.0 ± 73.1 | 0.0894 |  | 0.13144 ± 0.006 | 0.1298 ± 0.005 | 0.8420 |
| **GluD2** | **Ant** | 1988.4 ± 648.4 | 3033.2 ± 612.8 | 0.2753 |  | 0.1336 ± 0.03 | 0.2224 ± 0.08 | 0.3059 |
|  | **Pos** | 1804.4 ± 341.3 | 3300.4 ± 413.6 | 0.0236 ***** |  | 0.1196 ± 0.007 | 0.1602 ± 0.01 | 0.0423 ***** |
|  | **Floc** | 1708.8 ± 300.7 | 3611.2 ± 416.4 | 0.006 ****** |  | 0.116 ± .005 | 0.1902 ± 0.03 | 0.0271 ***** |

**Supplementary Media**

**Link to Z stack**

**References**

Celio, M. R., Baier, W., Scharer, L., Gregersen, H. J., de Viragh, P. A., & Norman, A. W. (1990). Monoclonal antibodies directed against the calcium binding protein Calbindin D-28k. Cell Calcium, 11(9), 599–602. https://doi.org/0143-4160(90)90014-L [pii]

Nakagawa, S., Watanabe, M., Isobe, T., Kondo, H., & Inoue, Y. (1998). Cytological compartmentalization in the staggerer cerebellum, as revealed by calbindin immunohistochemistry for Purkinje cells. The Journal of Comparative Neurology, 395(1), 112–120. Retrieved from http://www.ncbi.nlm.nih.gov/pubmed/9590549

Panzanelli, P., Früh, S., & Fritschy, J. M. (2017). Differential role of GABAA receptors and neuroligin 2 for perisomatic GABAergic synapse formation in the hippocampus. Brain Structure and Function, 1–13. https://doi.org/10.1007/s00429-017-1462-7

Knuesel, I., Mastrocola, M., Zuellig, R. A., Bornhauser, B., Schaub, M. C., & Fritschy, J. M. (1999). Altered synaptic clustering of GABA(A) receptors in mice lacking dystrophin (mdx mice). European Journal of Neuroscience, 11(12), 4457–4462. https://doi.org/10.1046/j.1460-9568.1999.00887.x

Jukkola, P., Guerrero, T., Gray, V., & Gu, C. (2013). Astrocytes differentially respond to inflammatory autoimmune insults and imbalances of neural activity. Acta Neuropathologica Communications, 1(1), 70. https://doi.org/10.1186/2051-5960-1-70

Nolte, C., Matyash, M., Pivneva, T., Schipke, C. G., Ohlemeyer, C., Hanisch, U. K., … Kettenmann, H. (2001). GFAP promoter-controlled EGFP-expressing transgenic mice: a tool to visualize astrocytes and astrogliosis in living brain tissue. Glia, 33(1), 72–86. Retrieved from <http://www.ncbi.nlm.nih.gov/pubmed/11169793>

Fukaya, M., Tsujita, M., Yamazaki, M., Kushiya, E., Abe, M., Akashi, K., … Sakimura, K. (2006). Abundant distribution of TARP ??-8 in synaptic and extrasynaptic surface of hippocampal neurons and its major role in AMPA receptor expression on spines and dendrites. European Journal of Neuroscience, 24(8), 2177–2190. <https://doi.org/10.1111/j.1460-9568.2006.05081.x>

Shimuta, M., Yoshikawa, M., Fukaya, M., Watanabe, M., Takeshima, H., & Manabe, T. (2001). Postsynaptic Modulation of AMPA Receptor-Mediated Synaptic Responses and LTP by the Type 3 Ryanodine Receptor. Molecular and Cellular Neuroscience, 17(5), 921–930. https://doi.org/10.1006/mcne.2001.0981

Nagy, G. G., Al-Ayyan, M., Andrew, D., Fukaya, M., Watanabe, M., & Todd, A. J. (2004). Widespread expression of the AMPA receptor GluR2 subunit at glutamatergic synapses in the rat spinal cord and phosphorylation of GluR1 in response to noxious stimulation revealed with an antigen-unmasking method. J Neurosci, 24(25), 5766–5777. https://doi.org/10.1523/JNEUROSCI.1237-04.200424/25/5766 [pii]

Konno, K., Matsuda, K., Nakamoto, C., Uchigashima, M., Miyazaki, T., Yamasaki, M., … Watanabe, M. (2014). Enriched Expression of GluD1 in Higher Brain Regions and Its Involvement in Parallel Fiber – Interneuron Synapse Formation in the Cerebellum, 34(22), 7412–7424. https://doi.org/10.1523/JNEUROSCI.0628-14.2014

Watanabe, M, Fukaya, M., Sakimura, K., Manabe, T., Mishina, M., & Inoue, Y. (1998). Selective scarcity of NMDA receptor channel subunits in the stratum lucidum (mossy fibre-recipient layer) of the mouse hippocampal CA3 subfield. Eur J Neurosci, 10(2), 478–487. Retrieved from http://www.ncbi.nlm.nih.gov/entrez/query.fcgi?cmd=Retrieve&db=PubMed&dopt=Citation&list_uids=9749710

Sabio, G., Arthur, J. S. C., Kuma, Y., Peggie, M., Carr, J., Murray-Tait, V., … Cuenda, A. (2005). p38γ regulates the localisation of SAP97 in the cytoskeleton by modulating its interaction with GKAP. The EMBO Journal, 24(6), 1134–1145. <https://doi.org/10.1038/sj.emboj.7600578>

Miura, E., Fukaya, M., Sato, T., Sugihara, K., Asano, M., Yoshioka, K., & Watanabe, M. (2006). Expression and distribution of JNK/SAPK-associated scaffold protein JSAP1 in developing and adult mouse brain. Journal of Neurochemistry, 97(5), 1431–1446. https://doi.org/10.1111/j.1471-4159.2006.03835.x

Miyazaki, T., Fukaya, M., Shimizu, H., & Watanabe, M. (2003). Subtype switching of vesicular glutamate transporters at parallel ® bre ± Purkinje cell synapses in developing mouse cerebellum, 17. https://doi.org/10.1046/j.1460-9568.2003.02698.x

Sperk, G., Schwarzer, C., Heilman, J., Furtinger, S., Reimer, R. J., Edwards, R. H., & Nelson, N. (2003). Expression of plasma membrane GABA transporters but not of the vesicular GABA transporter in dentate granule cells after kainic acid seizures. Hippocampus, 13(7), 806–815. https://doi.org/10.1002/hipo.10133
